# Imaging of α-Synuclein Aggregates in a Rat Model of Parkinson’s Disease Using Raman Microspectroscopy

**DOI:** 10.3389/fcell.2021.664365

**Published:** 2021-09-10

**Authors:** Fide Sevgi, Eva M. Brauchle, Daniel A. Carvajal Berrio, Katja Schenke-Layland, Nicolas Casadei, Madhuri S. Salker, Olaf Riess, Yogesh Singh

**Affiliations:** ^1^Department of Women’s Health, Research Institute for Women’s Health, Eberhard Karls Tübingen University, Tübingen, Germany; ^2^Natural and Medical Sciences Institute (NMI), Tübingen University, Reutlingen, Germany; ^3^Cluster of Excellence iFIT (EXC 2180) “Image-Guided and Functionally Instructed Tumor Therapies”, Eberhard Karls University Tübingen, Tübingen, Germany; ^4^Department of Medicine/Cardiology, Cardiovascular Research Laboratories, David Geffen School of Medicine at UCLA, Los Angeles, CA, United States; ^5^Institute of Medical Genetics and Applied Genomics, Eberhard Karls Tübingen University, Tübingen, Germany

**Keywords:** α-synuclein, Raman microspectroscopy, Parkinson’s disease, brain, enteric nervous system

## Abstract

A hallmark of Parkinson’s disease (PD) is the formation of Lewy bodies in the brain. Lewy bodies are rich in the aggregated form of misfolded α-Synuclein (α-Syn). The brain from PD patients can only be analyzed after postmortem, therefore, limiting the diagnosis of PD to the manifestation of motor symptoms. In PD patients and animal models, phosphorylated α-Syn was detected in the peripheral tissues including the gut, thus, raising the hypothesis that early-stage PD could be diagnosed based on colon tissue biopsies. Non-invasive marker-free technologies represent ideal methods to potentially detect aggregated α-Syn *in vivo*. Raman microspectroscopy has been established for the detection of molecular changes such as alterations of protein structures. Using Raman imaging and microspectroscopy, we analyzed the olfactory bulb in the brain and the muscularis mucosae of colon tissue sections of a human BAC-SNCA transgenic (TG) rat model. Raman images from TG and WT rats were investigated using principal component analysis (PCA) and true component analysis (TCA). Spectral components indicated protein aggregates (spheroidal oligomers) in the TG rat brain and in the colon tissues even at a young age but not in WT. In summary, we have demonstrated that Raman imaging is capable of detecting α-Syn aggregates in colon tissues of a PD rat model and making it a promising tool for future use in PD pathology.

## Introduction

Parkinson’s disease (PD) is the second most common disorder among neurodegenerative diseases with 6.1 million persons afflicted worldwide as estimated in 2016 (Alves et al., 2008; Dorsey et al., 2018). This disease burden is projected to have doubled in the past 25 years, whilst the number of older people did not increase in the same amount, indicating environmental factors could have an important role in PD progression (Dorsey et al., 2018). PD is manifested by the loss of neurons in the *substantia pars nigra compacta* with an increased neural loss of up to 70% by the time of death (Cookson, 2009). The presence of Lewy bodies (LB) represents the pathological hallmark of PD, as they are linked to the death of the dopamine-producing cells in the brain (Munishkina et al., 2003). The major component of LB is the filamentous inclusion protein α-Synuclein (α-Syn) (Kalia and Kalia, 2015). The accurate process of *in vivo* LB formation is not known so far. However, it is widely accepted that aggregation of α-Syn into soluble oligomers and then insoluble amyloid fibrils are the foundation of LB (Shahmoradian et al., 2019). During the aggregation process, phosphorylation is a usual characteristic as posttranslational phosphorylation of α-Syn is observed in 90% of misfolded proteins, while in cytosolic α-Syn, only 4% is phosphorylated (Kim et al., 2014). Even though α-Syn is an abundant protein in the brain, its exact function remains elusive in neuronal loss and their effective functions.

In its unfolded native form, α-Syn is a monomeric or intrinsically disordered protein (IDP) in neuronal cells and is a highly conserved protein mostly found in the presynaptic terminals of neurons and possibly in the nucleus (Li et al., 2001; Kalia and Kalia, 2015; Flynn et al., 2018). However, α-Syn embraces an α-helical nature upon engaging with lipid membranes and detergent micelles (Maiti et al., 2004; Mensch et al., 2017). Nuclear magnetic resonance (NMR) studies also demonstrated that the N-terminal region of the protein tended to form a stable α-helical secondary structures (Eliezer et al., 2001; Devitt et al., 2018). Furthermore, *in vitro* studies suggested that monomeric α-Syn normally consists mostly of α-helical (49%) and extended β-strand and polyproline II (PPII) structures (41%) with only a small amount of β-sheet present (10%) (Apetri et al., 2006). Transformation of α-helices by the increased presence of β-sheets was described earlier during α-Syn aggregation toward oligomers, protofibrils, and finally to the mature fibrils (Verma et al., 2015; Devitt et al., 2018). At the end of the fibrilization process, α-Syn mostly consists of well-ordered β-sheets (Flynn et al., 2018). In a more progressed fibrillization with protofibrils, β-sheets (54%) are the secondary structures in the majority, while the numbers of α-helical (37%) and extended β-strand and PPII (9%) structures are decreased (Apetri et al., 2006). While the mature fibrils are known to be toxic for cells, however, current evidence suggests that the intermediate species appears even more neurotoxic (Bengoa-Vergniory et al., 2017). Nonetheless, due to the sporadic nature of PD, many different forms of α-Syn aggregates may exist and thus, needs further investigation.

Many of the non-motor symptoms are associated with the impaired peripheral nervous system or the peripheral part of the central nervous system (vagus nerve, olfactory bulb, etc.) (Pellegrini et al., 2016; Liu et al., 2017). The receptor neurons of the olfactory bulb are exposed directly to the environment, giving an interface where environmental factors could trigger α-Syn aggregation (Liu et al., 2017). For α-Syn aggregation to occur, the enteric neurons (axons of myenteric plexus and/or submucosal plexus) need to be triggered by an intrinsic or environmental factor (Holmqvist et al., 2014). A recent study highlighted that enteroendocrine cells (EECs) were directly linked to enteric neurons, and therefore to the brain through the gastrointestinal muscles and the vagus nerve (Chandra et al., 2017). Furthermore, EECs also contained native α-Syn naturally, thus, these cells at the interface between environmental toxins and the enteric nervous system might be the source of the aggregation (Chandra et al., 2017; Liddle, 2018). Several earlier studies supported a notion of prion-like propagation of the α-Syn aggregation from cell to cell (Desplats et al., 2009; Visanji et al., 2013; Schweighauser et al., 2014; Liu et al., 2017; Candelise et al., 2019). Interestingly, α-Syn aggregates are also found inside gastrointestinal nerves, from the esophagus to the rectal end, before they can be observed in any of the dopamine-producing neurons (Lionnet et al., 2018). Direct transportation of α-Syn, injected into enteric neurons, toward the brain through the vagus nerve is demonstrated in several animal models; however, bidirectional propagation of α-Syn is possible (Uemura et al., 2018; Volpicelli-Daley and Brundin, 2018; Van Den Berge et al., 2019; Challis et al., 2020). Thus, peripheral tissues such as the colon could be an attractive target to detect the levels of α-Syn in PD patients or preclinical PD models.

Raman microspectroscopy is an ideal technology for use in medical and biochemical studies because of its high sensitivity and marker-free application (Brauchle and Schenke-Layland, 2013; Devitt et al., 2018). Raman spectra indicate changes of protein secondary structure based on specific peak shifts (Maiti et al., 2004). Previously, Raman microspectroscopy was utilized in a mouse model of Alzheimer’s disease (AD) for tau plaques from the brain (Michael et al., 2017; Ji et al., 2018). Furthermore, Raman spectroscopy has been used for the detection of α-Syn aggregations in *in vitro* studies (Mensch et al., 2017; Flynn et al., 2018). However, the use of Raman microspectroscopy for the detection of α-Syn in PD patients or preclinical rodent models has not been described so far to our knowledge. Thus, we hypothesized that Raman microspectroscopy could be utilized to recognize α-Syn forms, either native or aggregations, and their current structure in the fibrillization process to gain an insight into the progression of PD from the gut to the brain.

In this study, we used BAC-SNCA transgenic rats (called as TG) expressing full-length non-mutated human α-Syn (Nuber et al., 2013) and control wild-type (WT) rats at different ages [2–4 months (2–4M) and 12 months (12M)] to investigate the effects of aging and the differences between normal and pathological tissues due to expression of human α-Syn in the colon. Using Raman imaging and microspectroscopy together with immunofluorescence staining, we detected the presence of α-Syn-aggregated proteins and conformational changes in the protein secondary structures due to the fibrillization process in the brain and the colon tissues.

## Materials and Methods

### Animals Used for the Study

The BAC-SNCA transgenic (TG) rats were described earlier (Nuber et al., 2013) and corresponding age- and sex-matched wildtype (WT) rats (Sprague Dawley outbred genetic background) were used for this study. All the rats were kept in standard open-type IV cages (three to four rats/cage) under a 12-h light-dark cycle with *ad libitum* access to food pellet and water. All experiments were performed according to the EU Animals Scientific Procedures Act and the German law for the welfare of animals. All procedures were approved (TVA: HG3/18) by the authorities of the state of Baden-Württemberg, Tübingen, Germany.

### Colon and Brain Sample Preparation

The brain and colon tissues of WT and TG rats aged 4M or 12M were used for this study. The tissues were frozen in Tissue-Tek O.C.T (Sakura, Europe) compound and stored at −80°C until sectioning, and in some cases, formalin-fixed paraffin-embedded (FFPE) colon tissues were also used. Before sectioning, tissues were acclimatized at −20°C. A cryotome was used to cut tissue sections of 10 μm thickness, which were collected on standard glass slides. For the colon tissues, two to three sections per slide were collected, while for the brain tissues, one section per slide was collected. Twenty-five slides were collected for each sample. The remaining animal tissues were embedded into Tissue-Tek O.C.T (Sakura, Europe) compound for protection and transported at −80°C freezer. The tissue sections were stored in the −20°C freezer until further processing (details of chemical used in the study are available in Supplementary Table 1).

### Nissl Staining

Nissl staining was performed on selected brain tissues for differentiation of the different brain regions and to detect if the sections were intact enough for further processing. The sections were washed three times with DPBS for 5 min. Then, the tissues were fixed with 4% PFA for 15 min and washed again with DPBS for 15 min. The sections were then treated with 1% cresyl violet solution for 10 min followed by a few seconds in demineralized water (details of chemical used in the study are available in Supplementary Table 1). The slides were examined microscopically to ensure sufficient staining had occurred. Afterward, the samples were dehydrated analogously for H&E staining and washed twice with isopropanol for 5 min and mounted with isomount before being covered with a cover slip. The sections were then scanned with the slide scanner.

### Immunofluorescence Staining

Immunofluorescence staining was performed at least once per sample so that the α-Syn expressing regions could be identified. Primary and secondary antibodies were used for the staining (Supplementary Table 2).

The procedure of antibody staining was modified slightly from the protocol previously established (Brauchle et al., 2018). The sections were placed into racks and washed twice with DPBS for 10 min and fixed with 4% PFA for 20 min, before being washed again with DPBS twice for 5 min. The unspecific binding sites were blocked with goat blocking buffer. Next, the samples were treated with primary antibodies for an hour. After a washing step with the washing buffer, the samples were treated with the secondary antibody for 30 min in a dark room at room temperature (RT), followed by another washing step. If the samples were going to be measured directly with the Raman microspectrometer, the process was stopped, and the sections were stored in DPBS in a dark container for further use. If the samples were going to be just imaged with the fluorescence microscope, the samples were treated with DAPI for 10 min, and after a washing step, the sections were mounted with prolonged gold antifade mounting media (Thermo Fisher Scientific).

A control sample was always processed alongside the immunofluorescence staining for the evaluation of the staining success and the unspecific background staining. For the control samples, the previous procedure was performed with the exception that instead of diluting the primary antibodies in the dilution buffer, the dilution buffer was used on its own.

### Imaging of Immunofluorescence-Stained Sections

The sections stained with DAPI were examined with the observer fluorescence microscope (Zeiss GmbH, Germany). The 10 ×, 20 × and 40 × objectives were used, where with the 40 × immersion oil objective had to be applied to the samples. The microscopy was performed at a wavelength of 358 nm (DAPI, blue channel), 488 nm (green channel), and 594 nm (red channel). The software Zeiss Zen Blue Edition was used for the evaluation and processing of the images (Supplementary Tables 3, 4).

### Raman Imaging and Microspectroscopy

#### System Set-Up and Sample Preparation

A commercial Raman microspectroscope system (Alpha300R, WITec, Ulm, Germany) was used for all Raman measurements (Marzi et al., 2019; Zbinden et al., 2020). In brief, the system is equipped with a 532-nm laser, a filter to separate the scattered light and the excitation light, and a CCD camera (1,024 × 127 pixels) to detect the spectra. The samples were hydrated with DPBS and detected through a 63 × dipping objective with a numerical aperture of 1.0 (Zeiss, Oberkochen, Germany). The laser light was scattered from the samples and collected through the objective with CCD cameras. To eliminate any potential artefacts, the whole system was only used in a dark room and the CCD cameras were cooled to −60°C. Before any measurements could be taken, the performance of the Raman microspectroscope was verified by the measurement of a silicon wafer. The samples were excited with a laser power of 60 mW, and the scattered signal was analyzed with a grating of 1,800 g/mm centered at 1,300 rel. 1/cm. The microscope was also equipped with a filter set to visualize fluorescence-stained tissues. The biological samples examined were either untreated colon samples or immunofluorescence-stained colon or brain samples. All samples were kept in DPBS before and during the Raman measurements.

For each sample, three regions were selected from stained regions while the other three were selected randomly from non-stained regions of the brain samples. The large area scan width to height was 50 × 50 μm, the points per line and lines per image of the scan were 100 and 100, making the scan step size 0.5 μm. The integration time was selected as 0.5 s/pixel. A fluorescence image and a bright field image of the same area were overlapped to identify the immunofluorescence-stained area. In addition, single spectra were measured for later spectral analysis. In total, 12 single spectra were taken for the stained regions and the non-stained regions within one sample, with a total accumulation time of 100 s.

The untreated colon sections were measured by selecting three randomized areas in the muscularis externa. The large area scan width to height was 50 × 100 μm, the points per line and lines per image of the scan were 100 and 200, making the scan step size 0.5 μm. The integration time was 0.5 s. The immunofluorescence-stained colon sections were measured with the same parameters. A fluorescence image and the bright field image of the same sample area were overlapped to identify the stained area. Additionally, 15 single spectra were measured for each sample in stained regions for later analysis, with a total accumulation time of 100 s.

#### Pretreatment of the Spectra

For later statistical analysis, all spectra were pretreated with the Project 5.0 software (WITec, Ulm, Germany; Supplementary Table 4). Briefly, spectral data was automatically corrected for cosmic rays and the baseline corrected for each spectrum using a shape correction method with a shape size of 150 (WITec, Project 5.0). Shape correction means that the baseline is explained by circle and 150 corresponds to the size of each circle; these circles follow the baseline. The size can range from about 20 to 200 (minimum 20 and maximum 200). The baseline was similar in all spectra therefore, comparable. The spectra were then normalized, with the normalization type area to 1. Finally, the large area scans were stitched together so that later they could be analyzed together.

#### Raman Imaging and Spectral Analysis

Raman data were analyzed through different methods to detect differences in the samples at different time-points (4 M or 12 M) or of different genotypes (TG or WT).

The spectra (overall average of the Raman images of all the samples in each group) of the different groups (4M WT and TG and 12M WT and TG) were then compared with each other to identify differences. Peak positions, height, and full width at half maximum (FWHM) were automatically identified using the software-implemented peak listing (Project Five, WITec) The ratio of different peaks from amide I and amide III bands were compared. The compared peaks were related to changes of protein secondary structure: phenylalanine (1,004 cm^–1^)/amide III–β-sheet (1,267 cm^–1^), phenylalanine (1,004 cm^–1^)/amide III–α-helix (1,298 cm^–1^), phenylalanine (1,004 cm^–1^)/amide III–α-helix (1,340 cm^–1^), phenylalanine (1,004 cm^–1^)/amide I–α-helix (1,658 cm^–1^), amide III–β-sheet (1,267 cm^–1^)/amide III–α-helix (1,298 cm^–1^), amide III–β-sheet (1,267 cm^–1^)/amide III–α-helix (1,340 cm^–1^), and amide III–β-sheet (1,267 cm^–1^)/amide I–α-helix (1,658 cm^–1^). The statistical analysis through Student’s *t*-test was performed with Microsoft Excel 365.

#### Principal Component Analysis

Biological materials can produce complex datasets in Raman spectroscopy as several molecular vibrations produce overlapping peaks. Principal component analysis (PCA) is a multivariate analysis method that produces an optimized reduction of a spectral dataset to its principal components (PCs) is a well-established analysis tool used in Raman microspectroscopy (Pudlas et al., 2011). The first PC represents the highest amount of variation; the subsequent PCs refer to the next highest amount of variation chronologically. Score values represent the new variables of each spectrum and correspond to the calculated PCs (also called PC loading). PC score values were plotted against each other to visualize a correlation or separation of two or more datasets. The corresponding PC loading describes the spectral peak shifts that are responsible for the separation of a group.

In this study, PCA was performed on single spectra data from α-Syn-stained tissue regions using Unscrambler X 10.5 (Camo, Norway). Seven PCs were calculated for each analysis as described previously (Marzi et al., 2019; Zbinden et al., 2020). Score values from different groups were compared statistically with Student’s *t*-test using Microsoft Excel 365. The two PCs showing the highest statistically significant difference between the groups were presented in a score plot.

### Raman Image Analysis

All image analysis was conducted using Project FIVE Plus Software (WITec). The software-implemented “true component analysis (TCA)” was employed for spectral image analysis (Project Five Plus, WITec). In each spectral image, five component spectra were automatically identified, representing the major structures within the selected tissue site (collagen fibers, cell nuclei, muscle fibers, lipids, unknown component). This method delivers meaningful results in a fast and convenient way. In brief, it uses a linear combination of spectra (components) to describe each pixel of the image. Thus, spectra showing different information in an image can be separated, and based on the spectral pattern of the components, the composition of a sample can be identified (Marzi et al., 2019; Zbinden et al., 2020). For the colon samples, TCA analysis was performed of the whole area (intensity range of the pixels: 0–1) or of the stained regions (intensity range of the pixels: 0–0.8).

#### Statistics

For spectral analysis, PCA and TCA Raman images/scans were used (Supplementary Figures 1a,b; Supplementary Table 4). The samples were measured and separated into four groups: 4M WT, 4M TG, 12M WT, and 12M TG. The single spectra of the different groups were compared with each other to identify the difference between sample groups. Furthermore, the intensity and FWHM of the spectra were statistically analyzed with Student’s *t*-test or one-way ANOVA by employing the Kurskal Wallis with Tukey’s multiple comparison test. Data are presented as mean ± standard deviation (SD). Data with *p*-values ≤ 0.05 were identified as statistically significant.

## Results

### Identification of Endogenous α-Syn Aggregation in the Brain Olfactory Bulb Region of TG Rats

**FIGURE 1:**
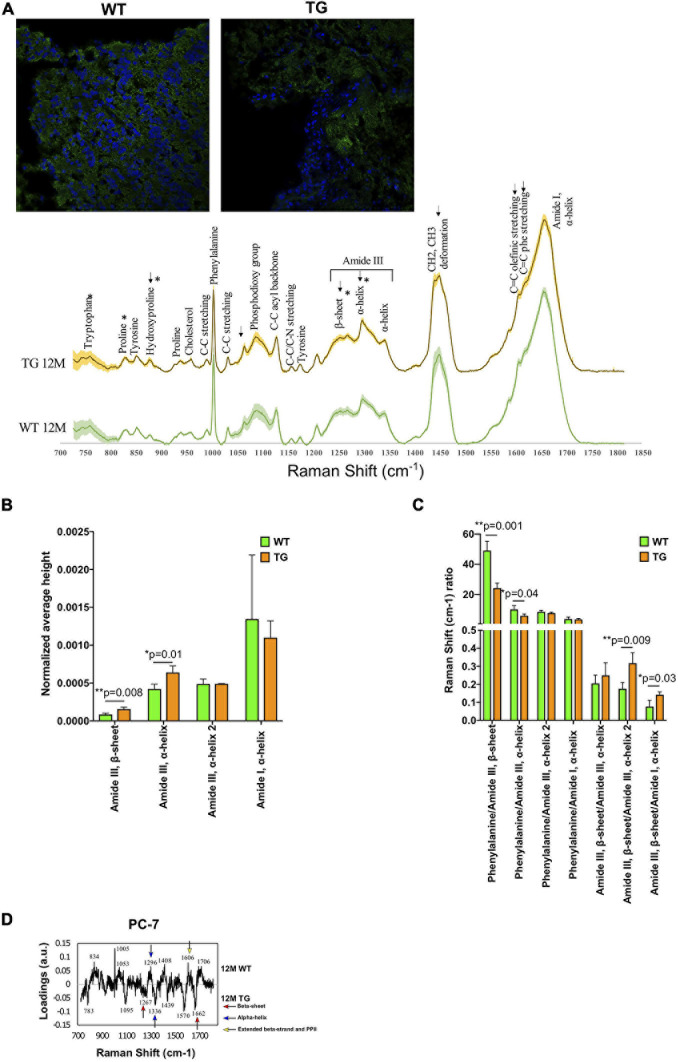
Spectral comparison in 12M WT and TG olfactory bulb regions. **(A)** Immunofluorescence images show the staining of α-Syn in the WT and TG olfactory bulb region of the brain (upper panel) and the spectra of WT and TG olfactory bulb regions (lower panel) and shaded areas indicate standard deviation. **(B)** All the marked changes were from the statistical 12M WT and TG intensity comparison based on Student’s unpaired *t*-test. Differences were detected in the tryptophan (*p* = 0.04), proline (*p* = 0.02), hydroxyproline (*p* = 0.04), amide III, β-sheet (*p* = 0.008), and amide III, α-helix (*p* = 0.01) peaks. *p*-Value represents *(*p* ≤ 0.05), **(*p* ≤ 0.01). **(C)** The ratio of different peaks from amide I and amide III were compared with Student’s unpaired *t*-test to identify statistical changes. Differences were detected in the phenylalanine/amide III–β-sheet (*p* = 0.001), phenylalanine/amide III–α-helix (*p* = 0.04), amide III–β-sheet/amide III–α-helix (*p* = 0.009), and amide III–β-sheet/amide I–α-helix (*p* = 0.03). *p*-Value represents *p ≤ 0.05 and **p ≤ 0.01. **(D)** The 12M WT and TG samples were compared with PCA of PC-6 and PC-7. The loadings of PC-7 were visualized. Positive side WT while negative side TG brain samples.

To identify the accumulation of α-Syn aggregation in the TG brain, we used the olfactory bulb brain regions as it is an early site of α-Syn accumulation (Niu et al., 2018; Stevenson et al., 2020). Initially, brain sections were used for Nissl staining for confirmation that the area of interest was suitable for the Raman measurements (Supplementary Figures 1a,b). Furthermore, we stained the samples for the endogenous rat-specific α-Syn antibody. We detected α-Syn staining faintly in the whole brain of either both genotypes WT or TG but predominantly on the edges (Figure 1A). Only the α-Syn-stained area was used for measurement by Raman microspectroscopy on a separate brain slide (Supplementary Figure 1b). After measurement of the samples, the assignment of peaks was identified based on literature (Supplementary Figure 1c and Supplementary Table 5).

**FIGURE 2:**
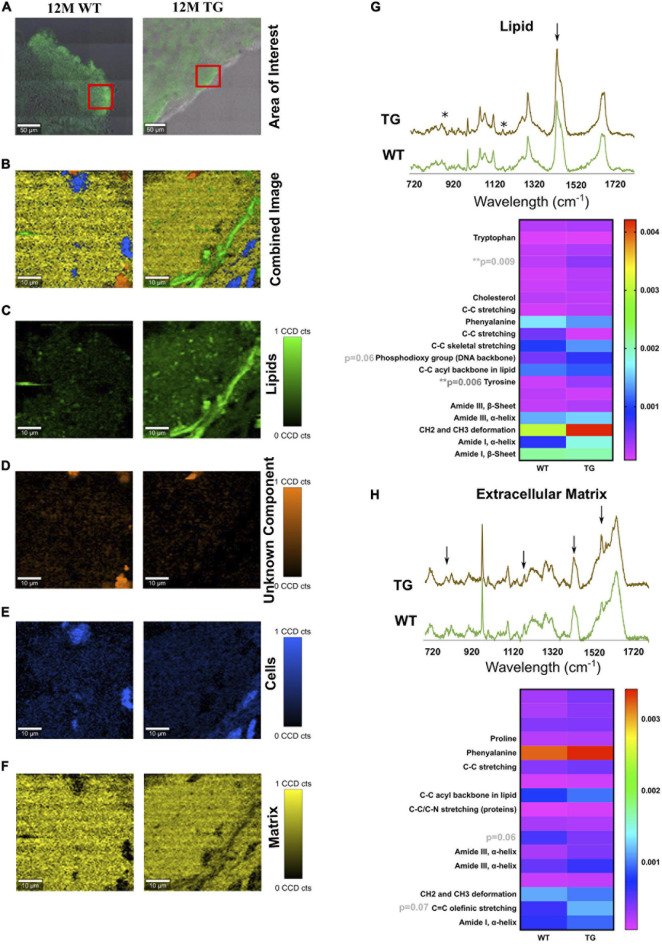
TCA of selected areas in the olfactory bulb of WT and TG animals. **(A)** The measured area is shown in red boxes. **(B)** The combined image of all the components is shown. **(C)** The separated components through TCA were lipids in green, **(D)** an unknown component in orange, **(E)** cells in blue, and **(F)** extracellular matrix components in yellow. The CCD count interval was listed for all the components. **(G**,**H)** The components displaying the same results were put together and averaged in their groups. The two components (lipids and extracellular matrix) out of five appeared to be different in most samples. *x*-axis represents the wavelength in centimeters while *y*-axis shows the spectral intensity. The separated groups were 12M WT (green) and 12M TG (brown). Differences in the lipid component were observed for intensity at unassigned peak 873 cm^–1^ (*p* = 0.009) and tyrosine (*p* = 0.006) between 12M WT and TG based on Student’s unpaired *t*-test. Heatmap represents the extracellular matrix component, some apparent difference in the spectra for unassigned molecule and C = C olefinic stretching; however, it did not reach a significant level. *p*-Value represents *p ≤ 0.05 and **p ≤ 0.01.

Univariate analysis was used to assess the statistical differences in the Raman spectra of WT and TG rats. The spectra of each Raman image were averaged (mean ± SD) and vector normalized. We compared Raman spectra of 12M WT and TG and showed signal patterns in the protein-rich structures. In 12M TG (*n* = 3), the rat brains had a higher intensity than the 12M WT (*n* = 4) rat brains except on the amide I shoulders, where WT rat brains have more relative intensity (Figure 1A). We found that the intensity of the tryptophan (759 cm^–1^) and proline (830 cm^–1^) was significantly lower in TG compared with WT rat brains, whereas hydroxyproline (877 cm^–1^), amide III, β-sheet (1,268 cm^–1^), and amide III, α-helix (1,298 cm^–1^) peaks were significantly higher in TG compared with WT rat brains (Figure 1B; Supplemnetary Figure 2). The Raman shift ratio was calculated when spectral peak data were normalized in the phenylalanine (1,004 cm^–1^) to amide III, β-sheet (1,267 cm^–1^) or amide III, α-helix (1,298 cm^–1^); a significant decrease in this ratio was noticed in TG compared with WT rat brains (Figure 1C). Significant higher expression of amide III β-sheets (1,267 cm^–1^) was discerned in TG rat brains in comparison with WT when spectral peak data were normalized to amide III, α-helix (1,340 cm^–1^) and amide I, α-helix (1,658 cm^–1^), indicating an increase of β-sheets relative to α-helix structures (Figure 1C). To sum up, our data suggests that Raman spectral signal patterns in the protein-rich structures in TG was clearly altered compared with WT rat brains.

The Raman spectra of 12M WT and TG brains were compared with each other through PCA to identify differences between the genotypes. A statistically significant separation was achieved in the molecular fingerprint region (800–1,800 cm^–1^) (Hashimoto et al., 2019) of the PC-6 and PC-7 score values (Supplementary Figure 3). Most of the WT samples were on the positive region, while most of the TG samples were on the negative region (Supplementary Figure 3). Two β-sheets were present in TG rats compared with WT, while WT rats had more α-helix, β-strand, and PP II than TG rat brains (Figure 1D). These results highlight the presence of aggregated α-Syn in the brain of TG rats.

The area of interest for Raman imaging was identified *via* α-Syn immunofluorescence staining. In Raman images from all samples, four major spectral components were identified: lipids, cell nuclei, matrix, and an unknown component (Figures 2A–F). No significant difference was observed in cell nuclei and the unknown component except that a significant difference in lipids was observed (Figures 2A–H). Lipids appeared to be more solid with less space in the TG rat brain compared with the WT brain samples (Figure 2C). Furthermore, in the lipid component, unassigned peaks near to hydroxyproline (873 cm^–1^) and tyrosine (1,175 cm^–1^) were significantly more intense (based on average height) in the TG brain samples compared with WT [Figure 2G, asterisks (^∗^)]. However, some visible differences were also observed in the phosphodioxy group (DNA backbone) (*p* = 0.06), CH2 and CH3 deformation peaks, though it did not reach a significance level (Figure 2G). Similarly, the protein (matrix) component in the brain region appeared to be different at the unassigned peak (1,209 cm^–1^) and C = C olefinic stretching (1,589 cm^–1^) peak (Figure 2H). Taken together, we concluded that TG rat brain samples had altered lipid and matrix components compared with WT brain samples.

### Label-Free Detection of α-Syn Aggregation in the Colon of TG Rats

**FIGURE 3:**
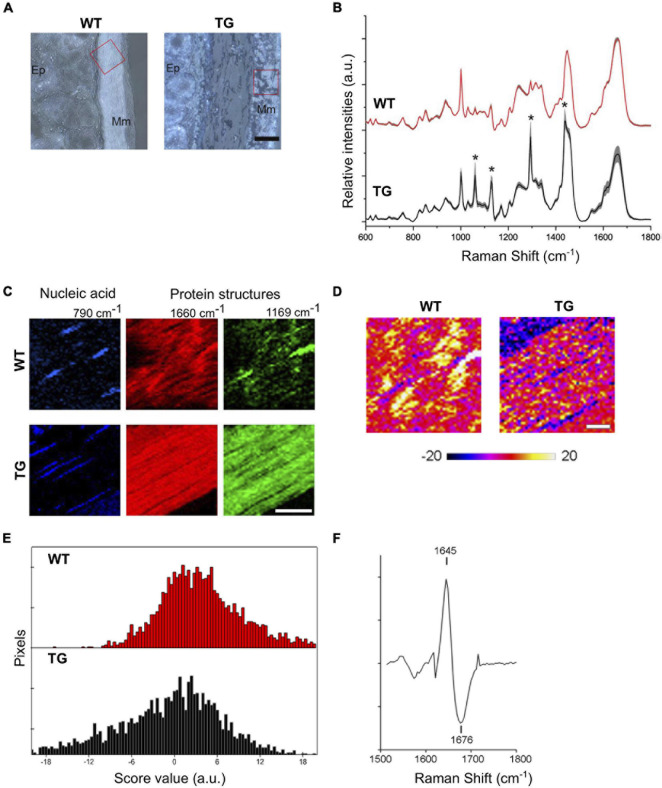
Label-free imaging of TG rat colons. **(A)** Bright field images of WT and TG rat colon tissues (2M). Scale bar equals 50 μm. Ep., epithelium; Mm, muscularis mucosae. Red box marks scanned areas using Raman microspectroscopy. **(B)** Mean Raman spectra of Raman images (*n* = 3). Grey shades indicate standard deviations. Asterisk indicates Raman peaks referring to paraffin. **(C)** Representative Raman images based on the sum intensities for specific peaks assigned to nucleic acids (790 cm^–1^) and proteins (1,660 and 1,169 cm^–1^) of WT and TG rat colon tissues. TG rat colons lack muscular striation pattern visible *via* amide I peak at 1,660 cm^–1^ in WT tissues. TG tissues show increased intensities for 1,169 cm^–1^ throughout the image. Scale bar equals 20 μm. **(D)** PCA of Raman images for amide I region indicate shifts in protein structure for TG rat tissues. **(E)** Representative images of WT and TG rat colon tissues of amide I region. Distribution of PC-2 score values showing range of positive values for WT and negative scores for TG tissues. **(F)** PC loading spectrum indicating a shift of amide I toward 1,676 cm^–1^ for pixels with negative score values.

After establishing the spectral pattern in the brain, we focused on the colon region with the predicted α-Syn aggregations that could be present in TG rats. First, we acquired the Raman spectra without labeling with α-Syn antibody from the FFPE tissues from WT and TG rats. Raman spectra also obtained from 2M WT (*n* = 3) and TG (*n* = 3) rat colon tissues showed signal patterns in the protein-rich structures (Figure 3). A bright field image of the colon tissue was shown (WT and TG) and red sqaure box highlighted area (mascularis mucosea layer) used for Raman spectra measurement (Figure 3A). Significant peaks were detected for proline (938 cm^–1^), phenylalanine (1,005 cm^–1^), C–C stretching (proteins) (1,169 cm^–1^), amide III (1,220–1,260 cm^–1^), and amide I (1,620–1,670 cm^–1^). Interestingly, Raman spectra of TG rats displayed strong contribution of C–O and C–C vibrational modes (1,060, 1,130, 1,300, and 1,437 cm^–1^) (Figure 3B), which were due to residual of paraffin wax in FFPE colon tissues and were thus not considered in this study. TG rat tissues showed lower relative intensities for proline, amide III and I, and α-helix, whereas signal intensities for C–C stretching (amino acids–tyrosine and phenylalanine) were increased in TG rat colon tissues compared with WT tissues (Figure 3B).

To visualize tissue structures of lamina muscularis mucosae in WT and TG colon, summarized intensities of Raman peaks for nucleic acids (760–790 cm^–1^) and protein structures (1,640–1,670 and 1,155–1,185 cm^–1^) were employed. Intensity-based images of nucleic acid peak at 790 cm^–1^ specify cell nuclei (Figure 3C). Amide I signal peak around 1,660 cm^–1^ represents muscular striation pattern in WT colon tissues, which were less clear in TG samples (Figure 3C). Only a few pixels within the lamina muscularis mucosae of WT tissues showed the Raman signal at 1,169 cm^–1^, whereas in the lamina muscularis mucosae of TG tissues, the peak at 1,169 cm^–1^ was very prominent throughout the colon tissue (Figure 3C). Previously, the amide I region was accredited for protein conformational change. Thus, the distribution of amide I peak positions was compared among WT and TG colon tissues. To resolve further the amide I peak signal, PCA was performed for the spectral range 1,500–1,800 cm^–1^. Spectral changes ascribed to the structural protein were identified in PC-2 (Figure 3D). Spectra from WT tissues show predominantly positive score values compared with Raman images from TG tissues, which exhibited negative score values for PC-2 (Figure 3E). Loading spectra of PC-2 indicates a shift of the amide I signal for WT tissues toward shorter wavenumber (1,647 cm^–1^) and TG tissues toward a higher wavenumbers of 1,676 cm^–1^ (Figure 3F). These data suggest the presence of β-sheet conformations in TG rat colon tissues, which have been seen to increase during α-Syn aggregation and fibril formation.

### Identification of Changes in the Colon Tissues of TG Rats by Raman Spectra Using a Labeled Method

**FIGURE 4:**
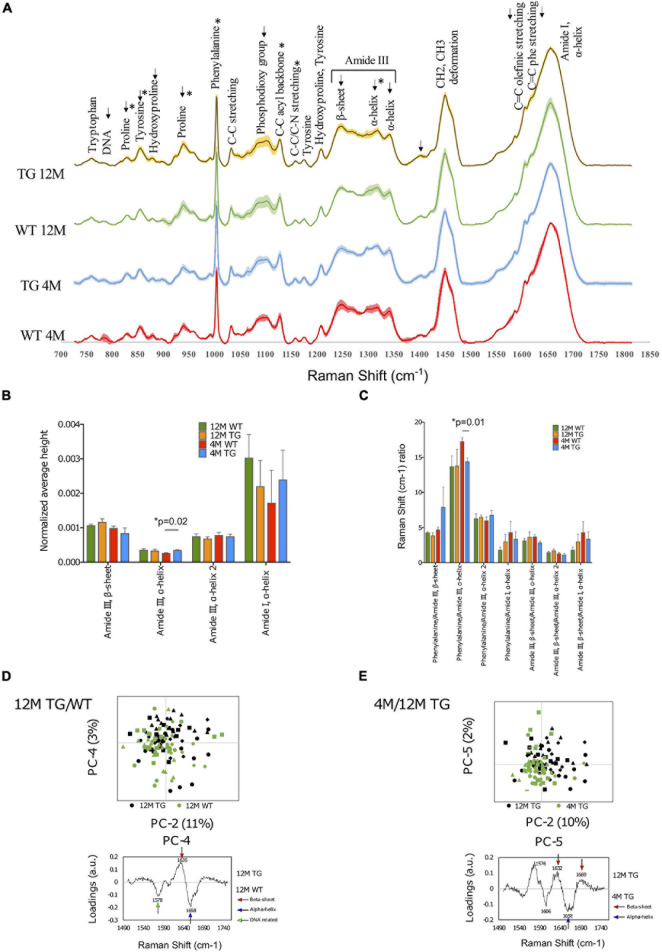
Spectral comparison at the genotype and age of the WT and TG rat colons. **(A)** The spectra of WT and TG colon samples at 4M and 12M age and shaded areas indicate the standard deviation. **(B)** Differences were detected at the intensity at 4M age for WT and TG in the amide III, α-helix (*p* = 0.01) peak. **(C)** The ratio of different peaks from amide I and amide III were compared with Student’s unpaired *t*-test to identify statistical changes. Differences were detected in the phenylalanine/amide III–α-helix ratio (*p* = 0.01). *p*-Value represents *p ≤ 0.05. **(D)** Comparison of the amide I 12M TG vs. WT samples through PCA with scores and loadings in colon. Amide I samples were compared with PCA at PC-2 and PC-4 (upper panel). PC-4 was significant (*p* = 0.01). The loadings of amide I PC-4 were visualized (lower panel). **(E)** Comparison of the amide I between 4M and 12M TG samples through PCA with scores and loadings in colon. Both PC-2 (*p* = 0.00003) and PC-5 (*p* = 0.004) were significant (upper panel). The loadings of amide I, PC-5 were visualized (lower panel).

Formalin-fixed paraffin-embedded tissues are not ideal for Raman spectral analysis due to additional paraffin peaks. Therefore, we focused our investigation on cryopreserved tissues obtained from WT and TG rats. We also stained the colon tissues with α-Syn antibody from freshly frozen tissue sections for gaining confidence on our data that we had obtained from label-free Raman imaging. The assignments of the peaks were identified which was comparable with the α-Syn antibody-stained brain samples (Figure 4A). For spectral analysis and PCA, single spectra were also measured while for TCA large area scans were used (Supplementary Figures 1c,d).

#### Spectral Analysis

First, we made the genotype comparisons between 4M WT (*n* = 3) and TG (*n* = 5) colon samples using spectral analysis (Figure 4B). However, only the intensity of the amide III, α-helix (1,317 cm^–1^) peak was statistically higher in 4M TG colon compared with WT (Figure 4B). When the phenylalanine peak was divided by the amide III peak, a significant difference was detected, and this peak ratio was found to be higher in WT compared with TG (Figure 4C). Furthermore, when 12M WT (*n* = 4) and TG (*n* = 4) samples were analyzed, visible differences were observed in the tyrosine (855 cm^–1^), hydroxyproline (879 cm^–1^), proline (938 cm^–1^), phosphodioxy group (1,096 cm^–1^), amide III, β-sheet (1,248 cm^–1^), amide III, α-helix (1,317 cm^–1^), and C = O stretch (1,402 cm^–1^) peaks, but it did not reach to a significant level between them (Figure 4A). These spectral data suggest that there were several changes in protein conformation in TG rat colon tissues.

Further aging comparisons were made among 4M and 12M TG colon samples. The 12M TG colon sample peaks were more intense in every marked area except for the proline (829 cm^–1^) peak and the two α-helix peaks of amide III (1,299–1,317 and 1,342 cm^–1^). Statistical differences were observed in the intensities of the proline (829 cm^–1^; lower), tyrosine (855 cm^–1^; higher), proline (939 cm^–1^; higher), phenylalanine (1,004 cm^–1^; higher), and C–C acyl backbone (1,158 cm^–1^; lower) peaks in 12M TG colon tissues compared with 4M TG (Supplementary Figure 4a). No statistical difference was observed in 4M and 12M WT rat colon samples (Figures 4A,C). Thus, our spectral data highlights that the aging process has an important change in amino acids, protein, and lipid components in colon tissue composition of the TG rats based on peak intensities.

#### PCA Analysis to Identify the Aggregation of α*-Syn* in the TG Colon

##### Genotype comparisons in WT and TG rats at either 4M or 12M

The PCA analysis was performed on the colon tissues, utilizing a similar procedure as that used for the brain tissue analysis of the molecular fingerprint region (800–1,800 cm^–1^). First, we calculated the PCA to identify the differences at 4M TG and WT colon tissues. Significant separation was observed at the PC-4 score values, whereas the other PCs did not show any significant differences. The PC-4 component was easily distinguishable in which 4M WT colon samples were mostly on the negative region while the 4M TG colon samples were mostly on the positive region (Supplementary Figure 4b). The most prominent peaks were phenylalanine (1,001 cm^–1^) and amide III, α-helix (1,321 cm^–1^) on the positive region for the 4M TG colon samples while DNA (787 cm^–1^) and T, A, G ring breathing modes of DNA/RNA bases (1,378 cm^–1^) on the negative region which represent 4M WT colon tissue samples. These data suggest the conformational change in amide III, α-helix in young (4M) TG rats compared with WT in the colon tissues which was stained for endogenous α-Syn.

In addition to the total Raman spectra (molecular fingerprint region), specific amide I and III regions were also subjected to PCA analysis for 4M TG and WT. In amide III, a significant separation was achieved through PC-1 score values (41% variance) and PC-4 score values (7% variance) (Supplementary Figure 4c). The loadings at PC-1 showed the α-helix (1,294 cm^–1^, 1,340 cm^–1^) on the positive region (mixed TG and WT) and the β-sheet (1,245 cm^–1^) with the shoulder α-helix (1,270 cm^–1^) on the negative region (WT). A significant separation was achieved through the PC-2 score values (8% variance) and the PC-7 score values (1% variance) in the amide I region (Supplementary Figure 4c). The loadings for PC-2 showed the β-sheet (1,637 cm^–1^) and β-sheet turn (1,685 cm^–1^) on the positive region and C = C olefinic stretch (1,586 cm^–1^) and C = C phenylalanine stretch (1,606 cm^–1^) on the negative region. The loadings for PC-7 showed the peaks β-sheet (1,630 cm^–1^) and α-helix (1,654 cm^–1^) on the positive region (WT) and β-sheet (1,638 cm^–1^) and extended β-strand and polyproline II (PPII) structures (1,670 cm^–1^) on the negative region (TG). Overall, based on the data, we concluded that 4M TG rat colon samples appeared to have a higher conformational change in the amide I region (C = C olefinic stretch, C = C phenylalanine stretch, β-sheet, extended β-strand, and PPII structures) compared with WT samples.

The 12M TG and WT samples were compared with each other through PCA to identify differences of modified rats at the same age (12M). PCA of fingerprint region did not show any difference among WT and TG rat samples. The amide I and amide III regions were analyzed closer with separate PCAs. In amide III, a significant separation was achieved through the PC-6 score values (1% variance); although the separation of PC-6 was overlapping among WT and TG, it was difficult to define the specific regions for WT and TG. The loadings of PC-6 showed β-sheet (1,262 cm^–1^) and α-helix (1,310 cm^–1^) on the positive region and α-helix (1,296 cm^–1^) on the negative region (Supplementary Figure 5a). In amide I, a significant separation was achieved through the PC-4 score values (3% variance) (Figure 4D). The separation at PC-4 was overlapping, but recognizable. The 12M TG samples were more on the positive region, while the 12M WT samples were more on the negative region (Figure 4D). The most significant PC-4 was visualized with PC-2 with their corresponding loadings (Figure 4D). The loadings of PC-4 showed the β-sheet (1,635 cm^–1^) on the positive region and the nucleic acids (1,578 cm^–1^) and α-helix (1,658 cm^–1^) peaks on the negative region (Figure 4D). These data suggest conformational change in amide I region of β-sheet in 12M TG rats compared with WT in colon tissues.

##### Aging comparisons in WT and TG rats at 4M and 12M

Furthermore, aging comparisons were made in either WT or TG rat (4M vs. 12M) samples through PCA of the fingerprint region to identify differences among either WT or TG colon samples with aging.

First, two WT samples at different ages (4M and 12M) were compared with each other through the fingerprint region of PCA to identify differences with the aging process in control (WT) rats. A significant separation was achieved in the PC-5 score values (6% variance), as the other PCs did not show any differences between 4M and 12M WT (Supplementary Figure 6). The most prominent peaks were DNA (786 cm^–1^), phosphodioxy group (1,094 cm^–1^), T, A, G (1,378 cm^–1^), and nucleic acids (1,576 cm^–1^) on the positive region and phenylalanine (1,001 cm^–1^), C–C acyl backbone (1,128 cm^–1^), and C = O stretch (1,405 cm^–1^) on the negative region. Most of the major peaks in the positive region were related to DNA. Furthermore, the amide I and III regions were analyzed closer in separate PCAs. In the amide III band, a significant separation was achieved with the PC-4 score values (6% variance) while all the other PCs did not show any differences between 4M and 12M WT.

Furthermore, aging comparisons were made among 4M and 12M TG colon samples through PCA of the fingerprint region. Significant separation was detected at the PC-2 score values (25% variance), at the PC-3 score values (15% variance), and at the PC-4 score values (11% variance) (Supplementary Figure 5b). The most significant PC-4 was visualized with PC-2 with their corresponding loadings (Supplementary Figure 5b); 12M TG samples were more on the positive region, and the 4M TG samples were more on the negative region. The separation at PC-2 was less clear but distinguishable with the 12M TG samples more on the positive region and the 4M TG samples more on the negative region. The major peaks on PC-2 were phosphate group (860 cm^–1^), proline (940 cm^–1^), amide III, β-sheet (1,248 cm^–1^), amide III, α-helix (1,653 cm^–1^), and amide I, turn (1,685 cm^–1^) on the positive region and C–C skeletal stretch (1,065 cm^–1^), phosphodioxy group (1,086 cm^–1^), C–C acyl backbone (1,130 cm^–1^), amide III, α-helix (1,296 cm^–1^), and CH_2_ and CH_3_ deformation (1,439 cm^–1^) on the negative region. The loadings of PC-4 contained the main peaks β-sheet (1,637 cm^–1^) and extended β-strand and PPII structures (1,673 cm^–1^) on the positive region (12M TG) and phenylalanine (1,001 cm^–1^) and amide III, α-helix (1,315 cm^–1^) on the negative region (4M TG) (Supplementary Figure 5b).

The amide I and amide III regions were analyzed further in detail with separate PCAs (Supplementary Figure 5c). In amide I, a significant separation was achieved through the PC-2 score values (10% variance), the PC-3 score values (6% variance), and the PC-5 score values (2% variance) (Figure 4E). The separation was clearer with PC-5, where the 12M TG samples were more on the positive region, while the 4M TG samples were mostly in the negative region. The loadings for PC-2 showed the major peaks β-sheet (1,634 cm^–1^), β-sheet (1,668 cm^–1^), and turn (1,683 cm^–1^) on the positive region and the peak C = C phenylalanine stretch (1,605 cm^–1^) on the negative region (Supplementary Figure 5c). The loadings for PC-5 showed the major peaks nucleic acids (1,576 cm^–1^), β-sheet (1,632 cm^–1^) and β-sheet (1,669 cm^–1^) on the positive region and C = C phenylalanine stretch (1,606 cm^–1^) and α-helix (1,658 cm^–1^) on the negative region (Figure 4E). In summary (Supplementary Table 6), 12M TG contained a higher amount of β-sheet compared with 4M TG, while 4M TG contained advanced intermediate oligomers and more α-helices.

#### TCA Analysis to Detect Other Cell Components With Aging in the TG Rat Colon

**FIGURE 5:**
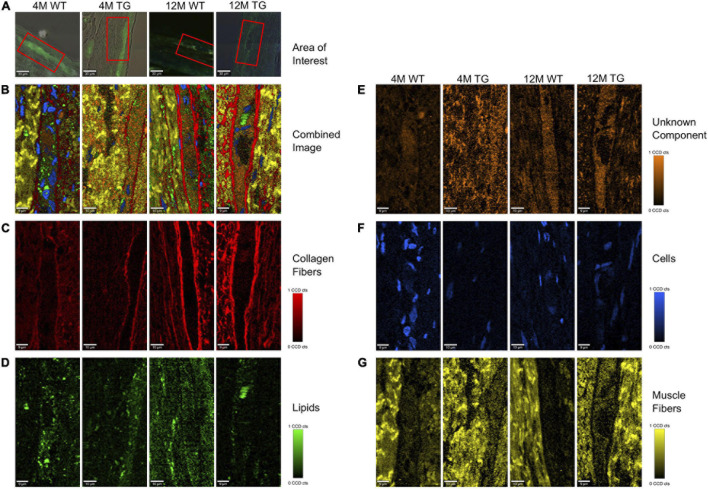
True component analysis (TCA) of selected areas in the colon of WT and TG rats. **(A)** The measured area is shown in red boxes. **(B)** The combined image of the components is shown. **(C)** The separated components through TCA were collagen fibers in red, **(D)** lipids in green, **(E)** unknown component in orange, **(F)** cells in blue, and **(G)** muscle fibers in yellow. The CCD count interval was listed for all the components.

Every measured sample with Raman microspectroscopy was also subjected to TCA. We stained the colon tissues with α-Syn staining (D37A6) antibody to achieve appropriate results; for the TCA, only the antibody-stained positively region was used (Figures 5A,B).

Raman images showed that collagen fibers surrounded the α-Syn-stained regions in all the samples with a more intense appearance in the older rats, presumably increasing in thickness with age (Figure 5C). Lipids appeared to be more concentrated in the stained regions with no changes in intensities between genotypes (Figure 5D). The colon unknown component appeared more solid in the α-Syn stained regions, possibly showing a connection with α-Syn protein. The intensity was lower in the 4M WT region, with no visible changes in the other groups (Figure 5E). The cells were mostly observed in the non-stained regions in all animals except the 4M WT sample, where cells were also observed inside the stained area (Figure 5F). The cells in the TG groups appeared smaller, possibly in the process of apoptosis. The muscle fibers were concentrated in the regions surrounding the staining, with no visible changes in intensity between the groups (Figure 5G). The spectral components of TCA were separated into their respective groups (4M WT, 4M TG, 12M WT, and 12M TG). The spectra highlighting the same component were averaged and graphed together so that the differences of the groups in the same component could be visualized (Supplementary Figure 7).

After the extraction of the different components through TCA (collagen fibers, lipids, unknown component, cells, and muscle fibers), the CCD counts and the number of pixels were scaled to an interval where all the positive pixels were contained. From the CCD count and the pixels, an intensity was calculated. The average intensity per pixel was taken for each component in each group to detect any difference between the components when their intensity in the TCA was compared. After statistical analysis, changes were observed in the muscle fibers between 4M and 12M TG, and nearly significant also among the 4M WT and TG colon samples (Supplementary Figure 7).

**FIGURE 6:**
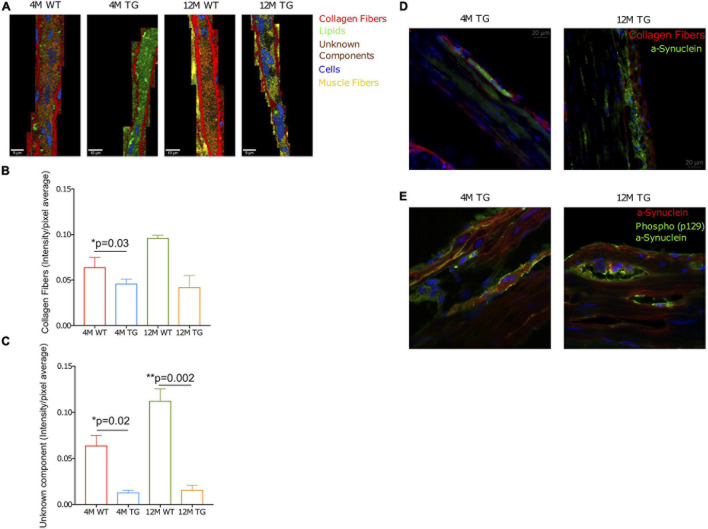
Collagen fibers, unknown component, and phospho-α-Syn in the TG rat colon. **(A)** Combined representative TCA images of the stained regions. The components were collagen fibers (red), lipids (green), an unknown component (orange), cells (blue), and muscle fibers (yellow). The intensity range was scaled 0–0.8 for all components. **(B,C)** The bar diagram showed the collagen intensity/pixel average for WT and TG rat colon samples at 4M and 12M age. Averaged intensities per pixel of the collagen fibers and unknown component statistically compared using Student’s unpaired *t*-test. *p*-Value represents *p ≤ 0.05. Significant differences were observed in the unknown component between 12M WT and TG (*p* = 0.002) as well as between 4M WT and TG 4M (*p* = 0.03). In contrary, statistical difference was observed in the collagen fibers only for 4M WT and TG (*p* = 0.02). *p*-Value represents *p ≤ 0.05 and **p ≤ 0.01. **(D)** Staining of the colon tissues with α-Syn (green, D37A6), collagen I (red, 113M4774) antibodies, and DAPI for cell nucleus for 4M and 12M TG rats. **(E)** The detection of phospho-α-Syn (p129; green) and α-synuclein (red, D37A6) and DAPI (blue) in the colon tissues of 4M and 12M TG rats.

The average intensities per pixel were taken for the components stained with α-Syn antibody (D37A6), in the regions where positive staining was observed. A significant difference was observed at 4M among WT and TG for collagen fibers (Figures 6A,B). Additionally, statistical differences were observed at the unknown component between 12M WT and TG, as well as those between 4M WT and TG colon samples (Figure 6C). Furthermore, the fluorescence microscope was used to display stained tissue sections for α-Syn and collagen fibers surrounding the α-Syn-stained regions (Figure 6D). In 12M TG, pockets between the α-Syn and collagen fibers were observed (Figure 6D). Cells (DAPI staining) were abundant in the surrounding regions but absent in the stained regions between adjacent collagen fibers (Figure 6D). In the 4M TG rats, no unstained areas were observed between the α-Syn and the collagen fibers. Morphologically, cells differed from the surrounding cells outside the stained region, being smaller in size and shorter in length (Figure 6D).

To understand a relationship between endogenous α-Syn and phosphorylation of α-Syn together with collagen fibers, we stained the TG colon tissues using phospho-α-Syn antibody (human) along with a total α-Syn protein (endogenous rat). This analysis revealed that in 12M TG colon tissue, phosphorylation/aggregation is mostly located in the pocket region compared with 4M TG rat colon samples (Figure 6E). Thus, altogether, these data explain that the pocket region between the total α-Syn and collagen regions could be due to aggregated α-Syn in older TG rats. Overall, our Raman imaging and microspectroscopy data revealed that aging is involved in the aggregation of α-Syn in the colon of TG rats.

## Discussion

Based on the notion at the beginning of the aggregation process in the colon, α-Syn protein molecules infect the neurons in a prion-like manner, propagating up the vagus nerve toward the midbrain, where it causes PD (Goedert, 2001; Kalia and Kalia, 2015; Volpicelli-Daley and Brundin, 2018; Challis et al., 2020). Therefore, aggregation of the proteins and changes in protein conformation was expected in the colon of younger rats, while progressed disease was probable in older TG rats. We confirmed the presence of aggregated proteins, detected changes in the secondary structure of the proteins due to the fibrillization process, and identified the changes in colon tissues through a combination of Raman imaging and Raman microspectroscopy.

In the brain, the olfactory bulb is one of the first regions where α-Syn aggregation was detected and accompanied by anosmia (Rey et al., 2016, 2018). After measuring all the samples, the fingerprint region was determined, and the peaks were assigned based on published literature (Supplementary Tables 5, 6). With Raman microspectroscopy, it was possible to image protein structures of native monomers, intermediate spheroidal oligomers, protofilaments, and fibrils. Most of these structures were obtained in the amide III (1,230–1,300 cm^–1^) and amide I (1,640–1,690 cm^–1^) regions (Maiti et al., 2004; Apetri et al., 2006). The spectra comparison of 12M WT and TG showed an increased intensity of β-sheet in TG brain samples. Furthermore, a significant difference was achieved for the β-sheet to the α-helix ratio in the amide III region. The fingerprint PCA resulted in peaks correlating with α-helix, extending β-strand and PPII in the 12M WT brain samples, whereas a higher content of β-sheets was detected in 12M TG rat brain samples (Figure 1D). In the amide III-specific PCA, only α-helices were detected in both the regions while, in the amide I-specific PCA, β-sheets were the dominating secondary structures in 12M TG brain samples. Thus, 12M TG rat brain contained dominantly β-sheet-rich secondary structures, signs of an advanced PD with protofibrils and mature fibrils, and in agreement with previously described *in vitro* findings (Celej et al., 2012). TCA analysis further revealed significant changes in lipid structures (tyrosine) suggesting that there were more solid lipid molecules in the TG compared with WT rat brain samples. Native α-Syn protein is known to bind naturally to lipids, making lipid molecules a reflection of α-Syn molecules (Munishkina et al., 2003). Changes in the secondary structures of lipids indicate an effect of α-Syn aggregation and points toward native α-Syn being present in the TG rats. TCA of the unknown components appeared to be changed but did not reach to a significant level in TG brain samples. Thus, the role of the unknown component could not be deciphered. Overall, the brain data suggest that signs of the fibrillization process was detected in 12M TG rats.

In the colon tissues, we first measured the change in the α-Syn aggregation using label-free and later endogenous α-Syn antibody staining methods as described in Alzheimer’s disease for misfolded amyloid-β protein using Raman microspectroscopy (Ji et al., 2018). Label-free imaging detected an increase in β-sheet conformations in TG rat colon tissues compared with WT samples. FFPE tissues were not an appropriate choice, therefore, further investigation was performed on cryopreserved tissues obtained from WT and TG rats. To have complete confidence of our results, we also stained the colon tissues with α-Syn antibody with freshly frozen tissue sections to get meaningful results as with label-free Raman imaging. No compelling signs of aggregation were observed in the WT samples at any age group (4M or 12M). It has been described previously that monomeric α-Syn tends to bind to DNA molecules, increasing the persistence length of the DNA (Jiang et al., 2018). Since most DNA-related peaks were correlating to the 4M WT samples and DNA binding was observed in monomeric α-Syn, thus, this could be taken as confirmation of the expected monomeric nature of the 4M WT samples. The amide III and amide I specific PCA confirmed the presence of DNA related peaks correlating with 4M WT on both bands. Additionally, α-helices were observed in both ages (4M and 12M) in WT colon, reinforcing the suspected monomeric nature of the WT at any given age samples as described earlier based on Raman spectra (Devitt et al., 2018).

Spectral analysis of the 4M WT and TG groups was performed for the identification of changes. In both the spectral comparison and its statistical analysis, a change between the groups was detected in the amide III–α-helix region (1,292–1,317 cm^–1^), where 4M TG colon was more intense compared with 4M WT colon samples. This observation was confirmed by the ratio comparisons of interesting peaks, where the phenylalanine to amide III–α-helix ratio was significantly increased in 4M TG. The fingerprint PCA analysis revealed that the corresponding DNA-related peaks in the 4M WT colon samples suggested the presence of monomeric α-Syn (Jiang et al., 2018). The amide I-specific PCA was mixed, therefore difficult to interpret, nevertheless, it was observed that 4M TG was coinciding dominantly with β-sheets. We assumed that the α-Syn in the 4M TG group was in the process of fibrillization into spheroidal oligomer or protofilament structures. As, only α-helices were observed, therefore, a complete fibrillization was not considered and 4M TG colon samples were possibly showing non-motor signs of PD that precede the diseases by decades, like constipation in the colon (Pellegrini et al., 2016; Sveinbjornsdottir, 2016; Bridi and Hirth, 2018).

Additionally, genotype comparisons among the 12M WT and TG colon samples were also examined. The comparison of the spectra revealed a change of intensity at the turning point from β-sheet to α-helix in the amide III region (Fink, 1998; Celej et al., 2012; Flynn et al., 2018). The intensity of the 12M TG samples were higher on the β-sheet region (1,248–1,262 cm^–1^) while the 12M WT samples gained intensity in the α-helix region (1,292–1,317 cm^–1^). Although, these results were not statistically significant except only for the C–C acyl backbone (1,128 cm^–1^) peak which was statistically significant among 12M WT and TG colon samples. PCA analysis revealed the separation in the amide I region, which was distinguished more easily as a β-sheet peak was detected in the 12M TG colon samples, while an α-helix peak was observed in the 12M WT colon samples. Based on these results, the 12M TG colon samples were assumed to have proceeded to a more advanced fibrillization process as only the β-sheets were observed in the correlating loadings, whereas the 12M WT colon samples contained an α-helix, indicating no advanced fibrillization (Apetri et al., 2006; Devitt et al., 2018).

The most interesting results were obtained when spectral comparison of 4M and 12M TG (genotype) colon samples were analyzed and we found that the turning point of β-sheet to α-helix in the amide III region, where the 12M TG colon samples appeared to have a higher intensity in the β-sheet region (1,248–1,262 cm^–1^) while the 12M WT colon samples gained intensity in the α-helix region (1,292–1,317 cm^–1^). However, the change was visible, and statistical analysis did not yield significant changes in the amide III region, and most statistical changes were present in the protein peak regions (829 to 1,004 cm^–1^). The fingerprint PCA resulted in three PCs, of which one was mixed (PC-3), while the other two (PC-2 and PC-4) could be allocated to specific regions. Two PC loadings showed β-sheets with higher intensity in the 12M TG group whereas the 4M TG colon samples contained mainly α-helices. The results indicated an advanced fibrillization process in the 12M TG colon samples as described earlier for *in vitro* studies using Raman microspectroscopy (Devitt et al., 2018).

The amide III-specific PCA was not clear as the groups were mixed. Nevertheless, β-sheet correlation to 12M TG was observed in several PC loadings. α-Helices, along with β-sheets on the 12M TG region were also observed with a higher number of β-sheets, indicating an advanced fibrillization. In the case of 4M TG colon samples, they were either allocated to both regions and did not present any peaks, thus no conclusion could be made. Furthermore, the amide I-specific PCA was clearer, where β-sheets were dominating in the 12M TG colon samples in most of the significant PC loadings. Along with the β-sheet, additional secondary structures were present to a lesser extent. The 4M TG colon samples contained both α-helices and β-sheets in equal numbers, suggesting advanced spheroidal oligomers or protofibrils due to the high β-sheet content (Apetri et al., 2006; Devitt et al., 2018). Considering that the 12M TG colon samples were aged further than the 4M TG colon samples, it would be plausible to assume they would be further advanced in the aggregation process, correlating with advanced PD with motor symptoms and possible dementia. Interestingly, the C–C phenylalanine stretch (1,606 cm^–1^) peak was correlating in all PC loadings of the 4M TG colon samples, indicating a connection, which has not been mentioned earlier in any related literature. The results were able to successfully confirm the presence of α-Syn aggregations in the colon enteric nervous system. Overall, our data highlights that 12M TG colon rats have an increased α-Syn with advanced fibrilization process.

The analysis of the different components in the colon samples (Brauchle and Schenke-Layland, 2013; Brauchle et al., 2018) revealed that the collagen fibers were higher in intensity in the 12M samples. The enclosing collagen fibers around the aggregation was pushed further from the surroundings in the 12M TG colon samples compared with the 4M TG, presumably increasing the thickness of the colon. Statistical comparison of the average intensity per pixel of the α-Syn-stained region displayed changes in collagen fiber intensity between 4M TG and WT, suggesting less collagen fiber composition in TG rat colons. However, with aging in TG colon samples (4M vs. 12M), it did not reach to significant levels, although, there was a tendency of increase in the colon level among WT from 4M to 12M. Further changes could be detected in the unknown component, where the intensity of the TCA image was noticeably increased in the α-Syn-stained area, enclosed by collagen fibers. This indicated that the unknown component of colon might be related to α-Syn. The statistical averaged intensity per pixel of the α-Syn area supported this concept as the intensity of the unknown component was significantly stronger in both the 4M WT and 12M WT samples compared with age-matched 4M TG and 12M TG samples, respectively, suggesting that the unknown component is associated with monomeric α-Syn molecules. There were empty pockets in the TG 12M sample, presumably left by the absence of the previously present cells. These results indicated a further advancement in the fibrillization process of TG 12M samples as the aggregation process was associated with cell death according to literature (Cookson, 2009; Desplats et al., 2009).

Changes were detected in the intensity of phenylalanine (muscle fibers) between 4M WT and TG comparisons, where the 4M WT intensity was higher compared with 4M TG. Furthermore, with aging in the TG rat colon, muscle fiber intensity is significantly increased (from 4M to 12M TG). No relationship could be found in literature between phenylalanine and α-Syn or muscle fibers. While the α-helix was more intense in the 12M WT samples compared with age-matched TG samples, indicating structural changes due to α-Syn expression. The average intensity per pixel of the muscle fibers was significantly higher intensity in the 4M WT compared with the 4M TG colon samples (Supplementary Figure 7f). These results indicate that there could be muscle damage in TG rat colon surrounding the α-Syn containing area.

Finally, we attempted to decipher a relationship between endogenous α-Syn and phosphorylation of α-Syn together with collagen fibers. We found that 12M TG rat colon tissue to have increased phosphorylation/aggregation in the pocket region compared with 4M TG rat colon samples and reduced collagen fibers. Thus, reduction in collagen fibers could be involved in the α-Syn aggregation process in the TG rat colon. However, further analysis is warranted to understand the exact role of muscle fibers, collagen fibers, and lipids in the α-Syn aggregation or misfolding process.

## Conclusion and Limitations

Overall, in the colon, no changes were detected between the WT samples (4M vs. 12M), as both only contained α-helices. In comparison between the 4M samples, signs of fibrillization were detected in the amide I region of TG 4M, where α-helix, β-sheet and extended β-strand, and PPII molecules were detected, indicating early spheroidal oligomers. Interestingly, 4M WT colon samples contained DNA-related peaks, which according to published literature (Jiang et al., 2018) indicate native monomeric structures. An advanced fibrillization process was detected in the 12M TG colon sample, where mainly β-sheets were observed, while in 12M WT only α-helices were detected. These results were confirmed by the 4M and 12M TG comparisons, where more β-sheets were observed in the 12M TG colon samples. It should be noted that the collagen fibers in the colon were surrounding α-Syn-stained areas in TCA, allowing a possible approach to detect α-Syn containing regions without staining. Additionally, a possible component for the natively monomeric structures was identified in the unknown components, as it was occurring significantly higher in the WT groups than TG groups. Similarly, like the colon, the brain showed signs of advanced fibrillization in the 12M TG rats, supported by component analysis and microscopy images.

Nevertheless, our study also had a few limitations as well. Human-specific α-Syn antibody (total and aggregation-specific) would have been preferred to refine results in TG rats. Additionally, all the unknown components could not be defined in the current study. More time points (early or late stages of disease) in the rats would be of advantage to pinpoint the starting point of the aggregations more accurately in the colon and brain. The measurement of several more brain regions, or even the vagus nerve, would be necessary to understand the further progression of the disease. In the future, Raman microspectroscopy could be a routine tool to detect PD disease in advance through analysis of colon biopsies with a considerably reduced misdiagnosis rate, leading to better care and quality of life of the patients.

## Data Availability Statement

The original contributions presented in the study are included in the article/Supplementary Material, further inquiries can be directed to the corresponding author/s.

## Ethics Statement

The animal study was reviewed and approved by HG3/2018. No patients or human data used in this study.

## Author Contributions

EB and YS did the study design, performed the research, managed the overall project involved in the entire study, analyzed the data, made the figures, and wrote the manuscript. FS performed the experiments, data analysis, made the figures, and wrote the manuscript. DC performed the experiments and data analyses. KS-L, NC, MS, and OR provided tools, data analyses, discussions, funding generation, and edited the manuscript. All authors contributed to the article and approved the submitted version.

## Conflict of Interest

The authors declare that the research was conducted in the absence of any commercial or financial relationships that could be construed as a potential conflict of interest.

## Publisher’s Note

All claims expressed in this article are solely those of the authors and do not necessarily represent those of their affiliated organizations, or those of the publisher, the editors and the reviewers. Any product that may be evaluated in this article, or claim that may be made by its manufacturer, is not guaranteed or endorsed by the publisher.
