# Supplementary material for: Imaging of α-Synuclein Aggregates in a Rat Model of Parkinson’s Disease Using Raman Microspectroscopy
Source: Front Cell Dev Biol. 2021 Sep 10;9:664365. doi: 10.3389/fcell.2021.664365 (PMC8461246; doi:10.3389/fcell.2021.664365)
Supplement: Supplementary file 1 [file Data_Sheet_1.PDF]

Imaging of α-Synuclein aggregates in a rat model of Parkinson's disease using Raman microspectroscopy

Fide Sevgi[1#], Eva M Brauchle[1,2,3#], Daniel A Carvajal Berrio[1,3], Katja Schenke-Layland[1,2,3,4], Nicolas Casadei[5], Madhuri S Salker[1], Olaf Riess[5], Yogesh Singh[1,5*]

[1]Department of Women's Health, Research Institute for Women's Health, Eberhard Karls Tübingen University, Tübingen, Germany
[2]NMI Natural and Medical Sciences Institute at the Tübingen University, Reutlingen, Germany
[3]Cluster of Excellence iFIT (EXC 2180) "Image-Guided and Functionally Instructed Tumor Therapies", Eberhard Karls University Tübingen, Tübingen, Germany
[4]Department of Medicine/Cardiology, Cardiovascular Research Laboratories, David Geffen School of Medicine at UCLA, Los Angeles, CA, USA
[5]Institute of Medical Genetics & Applied Genomics, Eberhard Karls Tübingen University, Tübingen, Germany

[#]Equal contributions

*Address for correspondence:

Dr Yogesh Singh
Institute of Medical Genetics and Applied Genomics,
Eberhard Karls Tübingen University,
Calwerstaße 7, 72076, Germany
Phone: 0049 7071 29 78264
Email: yogesh.singh@med.uni-tuebingen.de

Suppl. Table 1: List of chemicals used for the study

Chemical	Company	Order Number
Bovine Serum Albumin	Sigma Aldrich (Darmstadt, GER)	A9576
Cresyl Violett Acetate	Waldeck (Münster, GER)	1A-400
DAPI	Sigma Aldrich (Darmstadt, GER)	D9542
Dulbecco's Phosphate-Buffered Saline (DPBS)	Gibco (Thermo Fisher Scientific) (Waltham, MA USA)	14190-169
Eosin G-Solution 1 % aqueous	Carl Roth (Karlsruhe, GER)	3137.2
Ethanol (EtOH) absolute	AppliChem (Darmstadt, GER)	A367812
Gelatin from cold water fish skin	Sigma Aldrich (Darmstadt, GER)	G7041
Goat Serum	Vector Laboratories (Burlingame, CA, USA)	S-1000-20
Hematoxylin solution according to Mayer	Carl Roth (Karlsruhe, GER)	T865.1
Isomount 2000	VWR (Darmstadt, GER)	05547535
Isopropanol (2-propanol)	Honeywell (Morris Plains, NJ, USA)	563935-1L
Paraformaldehyde (PFA) 4%	Sigma Aldrich (Darmstadt, GER)	15812-7
ProLong Gold Antifade Mountant	Invitrogen (Thermo Fisher Scientific) (Waltham, MA USA)	P36934
Tissue-Tek O.C.T.	Sakura Finetek (Torrance, CA, USA)	4583
Triton X-100	Sigma Aldrich (Darmstadt, GER)	T9284
Tween 20	Sigma Aldrich (Darmstadt, GER)	P1379

Suppl. Table 2: List of antibodies used for the study

Name	Dilution	Company	Order Number
Primary Antibodies			
α-Synuclein anti-Mouse Rabbit IgG	1:100	Cell Signaling Technology (Cambridge, GB)	D37A6
Collagen I A1 Mouse IgG1	1:800	Novusbio (Centennial, CO, USA)	113M4774
Secondary Antibodies			
AlexaFluor® 488 goat anti-Rabbit IgG		Invitrogen (Thermo Fisher Scientific) (Waltham, MA USA)	

	1:250		A-11034
AlexaFluor® 488 goat anti-Mouse IgG1	1:250	Invitrogen (Thermo Fisher Scientific) (Waltham, MA USA)	A-21121
AlexaFluor® 594 goat anti-Mouse IgG1	1:250	Invitrogen (Thermo Fisher Scientific) (Waltham, MA USA)	A-21125
AlexaFluor® 594 goat anti-Rabbit IgG	1:250	Invitrogen (Thermo Fisher Scientific) (Waltham, MA USA)	A-11037

Suppl. Table 3: List of instruments used for data acquisition

Equipment	Company
OpticLab H850 Slide Scanner	plustek (Taipeh, TWN)
Axio Observer microscope Z1	Zeiss (Oberkochen, GER)
Microscopy fridge	Liebherr (Bulle, CH)
Raman micro-spectroscope	WITec (Ulm, GER)
Laser scanning microscope	Zeiss (Oberkochen, GER)
Microm HM 560 Cryostat	Thermo Fisher Scientific (Waltham, MA, USA)
Microscope	Zeiss (Oberkochen, GER)
Suction device	neoLab (Heidelberg, GER)

Suppl. Table 4: List of software used for data analysis

Software	Developer	Application
Control Five	WITec (Ulm, GER)	Raman data acquisition
Project Five 5.2	WITec (Ulm, GER)	Raman data processing
Zeiss (Zen)	Zeiss (Oberkochen, GER)	Microscope imaging program
ZEN 3.0 (blue edition)	Zeiss (Oberkochen, GER)	Microscope image processing
Microsoft Excel	Microsoft Corporation (Redmond, WA, USA)	Data analysis and processing
Microsoft Word	Microsoft Corporation (Redmond, WA, USA)	Text processing
The Unscrambler X 10.5	CAMO Software (Oslo, NOR)	data analysis
GraphPad Prism 6	GraphPad Software (San Diego, CA, USA)	Data analysis and processing
TeamViewer 14	TeamViewer (Göppingen, GER)	Establishing connection from distance

Supp. Table 5: Identification of the Raman spectra

The assigned spectra were listed along with their literature source

SN	Wave number (cm^{-1})	Assignment	References

1	759	Tryptophan	(1, 2)
2	783	DNA	(1, 3)
3	790	O-P-O stretching DNA	(1, 4)
4	804	Uracil-based ring breathing mode	(1)
5	827	Proline	(1)
6	855	Tyrosine	(1)
7	860	Phosphate group	(1)
8	879	Hydroxyproline	(1)
9	938	Proline	(1)
10	957	Cholesterol	(1)
11	990	C-C stretching	(1)
12	1001	Phenylalanine	(1)
13	1032	C-C stretching	(1)
14	1053	C-O stretching	(1, 5)
15	1063	C-C skeletal stretching	(1)
16	1087-1099	Phosphodioxy group (DNA backbone)	(1)
17	1129	C-C acyl backbone in lipid	(1)
18	1156	C-C/C-N stretching (proteins)	(1)
19	1174	Tyrosine	(1)
20	1206	Hydroxyproline, Tyrosine	(1)
21	1248-1263	Amide III, β-sheet	(6-10)
22	1274	Amide III, α-helix	(11)
23	1280	Amide III, α-helix	(1, 2)
24	1290-1317	Amide III, α-helix	(1, 12)
25	1336-1342	Amide III, α-helix	(10, 13)
26	1378	T, A, G (ring breathing modes of the DNA/RNA bases)	(1)
27	1402	C=O stretching	(1)
28	1416	C=C stretching in quinoid ring	(1)
29	1440-1451	CH_2 and CH_3 deformation	(1)
30	1488	Nucleic acid purine bases	(1)
31	1570-1579	Nucleic acids	(1)
32	1586	C=C olefinic stretching	(1)
33	1592	C=N and C=C stretching in quinoid ring	(1)
34	1606	C=C phenylalanine stretching	(1)
35	1632-1637	β-sheet	(8, 9, 14)
36	1650-1658	Amide I, α-helix	(15, 16)
37	1664-1669	Amide I, β-sheet	(10, 14)
38	1670-1680	Amide I, extended β-strand and polyproline II (PPII) structure	(1, 14, 17)
39	1685	Amide I, β-turn	(1, 14, 18)
40	1706	C=O stretching	(1)

Suppl. Table 6: Overview of all the performed PCA and resulted peaks. The region in the PCA was shown in (+) or (-) next to the group. A star (*) next to a peak showed more intense peaks. The normalized region correlated to the fingerprint region.

Sample Type	Comparison	Region	Groups	DNA-related	β-sheet	α-helix	PPII	Turn	C=N, C=C	C=C phenyl-alanine
Brain	12 M WT vs. TG	Norm (PC6)	12M WT, 12M TG (+)							
			12M TG (-)	790*		1295*				
		Norm (PC7)	12M WT (+)			1296				1606
			12M TG (-)	783, 1570, 1095	1267, 1662	1336				
		Amide III (PC-5)	12M TG (+)			1294				
			12M WT (-)			1312				
		Amide I (PC-2)	12M TG (+)		1665					
			12M WT (-)							
		Amide I (PC-5)	12M TG (+)	1570	1669					
			12M WT (-)			1657				1605
Colon	4M vs. 12M WT	Norm (PC5)	4M WT (+)	786, 1378, 1576, 1094						
			12M WT (-)							
		Amide III (PC-4)	12M WT (+)			1283, 1320				
			4M WT (-)	1377*		1297, 1342				
		Amide I (PC-3)	12M WT (+)			1654				
			4M , 12M WT (-)	1578						
	4M WT vs. TG	Norm (PC4)	4M TG (+)			1321*				
			4M WT (-)	787*, 1378						
		Amide III (PC-1)	4M TG, WT (+)			1294*, 1340				

			4M WT (-)		1245**	1270*				
		Amide III (PC-4)	4M TG, WT (+)			1287, 1317				
			4M WT (-)	1378*		1341				
		Amide I (PC-2)	4M WT (+)		1637			1685*		
			4M TG, WT (-)							1606
		Amide I (PC-7)	WT 4M (+)		1630	1654				
			TG 4M (-)		1638, 1670					
	12M WT vs. TG	Amide III (PC-6)	12M TG, 12M (+)		1262	1310				
			12M TG, WT (-)			1296				
		Amide I (PC-4)	12M TG (+)		1635*					
			12M WT (-)	1578		1658*				
	4M vs. 12M TG	Norm (PC2)	12M TG (+)		1248*	1653		1685		
			4M TG (-)	1086*		1296				
		Norm (PC3)	4M, 12M TG (+)	1100						
			4M, 12M TG (-)			1293				
		Norm (PC4)	12M TG (+)		1637		1673			
			4M TG (-)			1315*				
		Amide III (PC-1)	4M, 12M TG (+)			1312, 1340				
			12M TG (-)		1247*	1273				
		Amide III (PC-2)	4M,12M TG (+)							
			12M TG (-)		1250	1312, 1340*				

		Amide III (PC-5)	4M TG (+)							
			12M TG (-)			1285, 1347				
		Amide I (PC-2)	12M TG (+)		1634, 1668			1683 *		
			4M, 12M TG (-)							1605
		Amide I (PC-3)	TG 4M, TG 12M (+)	1580*	1668*					1606*
			TG 12M (-)			1647* *				
		Amide I (PC-5)	TG 12M (+)	1576*	1632					
			TG 4M (-)		1669*	1658*				1606

Suppl. methods:

Hematoxylin and Eosin staining

Hematoxylin and eosin staining (H&E) was performed on selected colon tissues for morphological evaluation of the tissue sections and the identification of the area of interest. The tissue sections were washed three times with DPBS for 5 minutes, then, fixed with 4% PFA for 15 minutes and then washed again with DPBS for 15 minutes. The sections were then treated with hematoxylin solution for 9 minutes and afterwards with demineralized water for a few seconds and examined microscopically if the staining was sufficient. Next, the sections were left under running warm water for 10 minutes to wash away excessive staining and briefly washed with demineralized water for a few seconds. Afterwards, the sections were treated with eosin solution for 2 minutes, before being treated with demineralized water again for a few seconds. The samples were then put through a dehydration procedure of 70% ethanol (EtOH), 90% EtOH and 100% EtOH for 5 minutes each (details of chemical used in the study are available in Suppl. Table 1). Next, the sections were washed twice with isopropanol for 5 minutes. The sections were then mounted with isomount and covered with a thin glass slide. Later the sections were scanned with the slide scanner.

Suppl. Figure legends

Suppl. Figure 1. Identification of the area of interest in the brain sample. (a) Representative Nissl-stained brain section with the black square identifying the olfactory bulb area, also observed in the laser scanning microscope LSM. (b) Alpha-synuclein stained (D37A6) brain section with olfactory bulb. The area was visualized with the LSM. (c) Representative example of single spectrum scans in brain with antibody staining (D37A6) as shown in b. Single spectra measurements were depicted as crosses and large are scans as squares in the stained regions. Single spectra were used in spectral analysis and PCA analysis. The Raman spectra were identified based on different sources in literature. The exemplary spectrum was taken from WT 12M. (d) Identification of the Area of Interest in the colon sample WT 4M. H&E-stained colon

section with the black square identifying the area in the 63x magnification. Magnification of 10x, pictured through the Raman microspectroscopy with the black square identifying the area in the 63x magnification. Magnification of 63x, pictured through the Raman microspectroscopy with the red square identifying the measured area with a large area scan. The layers of the colon tissue were identified as ME: Muscularis Externa, SM: Submucosa, MM: Muscularis Mucosa and E: Epithelial cells. Representative example of single spectrum scans with antibody staining (D37A6) in colon (right hand side image). Single spectra measurements were depicted as crosses in the most stained regions and used in spectral analysis and PCA analysis.

Suppl. Figure 2. Spectral comparison in the olfactory brain regions. (a) The WT 12M and TG 12M samples were compared through their mean and standard deviation. The intensities of the spectra were statistically analyzed through a t-test to detect any statistical changes. Differences were identified in the 759 cm-1 (p=0.04), 830 cm-1 (p=0.02), 877 cm-1 (p=0.04), 1268 cm-1 (p=0.008) and 1298 cm-1 (p=0.01) peaks.

Suppl. Figure 3. Comparisons of PCA for genotyping in the rat brain region. Comparison of 12M WT and TG samples through PCA with scores and loadings in brain. Both PC-6 (p=0.03) and PC-7 (p=0.006) were significant (upper panel). The loadings of PC-6 were visualized.

Suppl. Figure 4. Comparisons of spectra and PCA for genotyping and ageing in the rat colon region. (a) FWHM spectra for 12M WT and TG colon samples. (b) Ageing comparison (4M vs 12M TG) of Raman spectra for normalized average height. (c) Comparison of 4M WT and TG samples through PCA with scores and loadings in colon. The 4M WT and TG samples were compared with PCA at PC-4 and PC-2. PC-4 was significant (p=0.001). The loadings of PC-4 were visualized. (d) Comparison of the amide III and amide I 4M WT and TG samples through PCA with scores and loadings in colon. The 4M WT and TG amide III samples were compared with PCA at PC-1 and PC-4. Both PC-1 (p=0.003) and PC-4 (p=0.01) were significant. The loadings of amide III, PC-1 and PC-4 were visualized. The 4M WT and TG amide I samples were compared with PCA at PC-2 and PC-7. Both PC-2 (p=0.03) and PC-7 (p=0.003) were significant. The loadings of amide I, PC-2 and PC-7 were visualized.

Suppl. Fig. 5. Comparisons of PCA for genotyping (12M WT and TG) and ageing (3M-12M TG) in the rat colon region. (a) Comparison of the amide III 12M TG and WT samples through PCA with scores and loadings in colon. The 12M TG and WT amide III samples were compared with PCA at PC-5 and PC-6. PC-6 was significant (p=0.03). The loadings of amide III, PC-6 were visualized. (b) The 4M and 12M TG samples were compared with PCA at PC-2 and PC-4. Both PC-2 (p=0.008) and PC-4 (p=0.0004) were significant. Furthermore, 4M and 12M TG samples were compared with PCA at PC-3 and PC-4. Both PC-3 (p=0.03) and PC-4 (p=0.0004) were significant. The loadings of PC-2, PC-3 and PC-4 were visualized. (c) Comparison of the amide III and amide I 4M and 12M samples through PCA with scores and loadings in colon. 4M and 12M TG amide III samples were compared with PCA at PC-1 and PC-5. Both PC-1 (p=0.0003) and PC-5 (p=0.0007) were significant. 4M and 12M TG amide III samples were compared with PCA at PC-1 and PC-2. Both PC-1 (p=0.0003) and PC-2 (p=0.04) were significant. The loadings of amide III, PC-1, PC-2 and PC-5 were visualized. Furthermore, 4M and 12M TG amide I samples were compared with PCA at PC-2 and PC-3. Both PC-2 (p=0.00003) and PC-3 (p=0.03) were significant. Additionally, 4M and

12M amide I TG samples were compared with PCA at PC-2 and PC-5. Both PC-2 (p=0.00003) and PC-5 (p=0.004) were significant. The loadings of amide I, PC-2, PC-3 and PC-5 were visualized.

Suppl. Fig. 6. Comparisons of PCA for ageing (4M-12M TG) in the rat colon region. (a) The WT 4M and 12M samples were compared with PCA at PC-5 and PC-6. PC-5 was significant (p=0.03). The loadings of PC-5 were visualized. (b) The WT 4M and 12M amide III samples were compared with PCA at PC-4 and PC-7. PC-4 was significant (p=0.03). The loadings of amide III, PC-4 were visualized. The WT 4M and 12M amide I samples were compared with PCA at PC- 3 and PC-5. PC-3 was significant (p=0.03). The loadings of amide I PC-3 were visualized.

Suppl. Fig. 7. TCA components in colon viewed in different groups. The components displaying the same results were put together and averaged in their groups. The five components calculated in all animal samples were collagen fibers, lipids, unknown component, cells and muscle fibers. The separated groups were WT 4M (red), TG 4M (blue), WT 12M (green), TG 12M (brown). Statistical ANOVA analysis was performed for intensity and FWHM and the significant peaks were presented in black squares. (a) Differences in collagen fibers were observed for intensity at 1245 cm-1 between WT 4M and 12M (p<0.0001) and WT and TG 12M (p<0.0001), and for FWHM at 759 cm-1 between TG 4M vs. 12M (p=0.02), at 1245 cm-1 between WT 4M vs. 12M (p=0.01) and WT vs. TG 12M (p=0.004), and at 1668 cm-1 between WT 4M vs. 12M (p=0.002). (b) Differences in lipids were observed for intensity at 1257 cm-1 between WT 4M vs. 12M (p<0.0001) and WT vs. TG 12M (p<0.0001), and for FWHM at 851 cm-1 between WT vs. TG 12M (p=0.03). (c) Differences in the unknown component were observed for intensity at 1004 cm-1 between TG 4M vs. 12M (p=0.02), and for FWHM at 1313 cm-1 between WT vs. TG 4M (p=0.0008). (d) Differences in cells were observed for intensity at 786 cm-1 between TG vs. WT 4M (p=0.04) and at 1665 cm-1 between TG vs. WT 4M (p=0.0006), and for FWHM at 1665 cm-1 between TG 4M vs. 12M (p=0.01). (e) Differences in muscle fibers were observed for intensity at 1004 cm-1 between TG vs. WT 4M (p<0.0001) and TG 4M vs. 12M (p=0.0002) and at 1653 cm-1 between TG vs. WT 12M (p=0.03). (f) Averaged intensity per pixel of muscle fibers statistically compared through t-test. The four groups were statistically compared through t-test. Differences were observed in muscle fibers between TG 12M vs. TG 4M (p=0.04) and TG 4M vs. WT 4M (p=0.057).

References

1. Z. Movasaghi, S. Rehman and I. U. Rehman: Raman Spectroscopy of Biological Tissues. Applied Spectroscopy Reviews, 42(5), 493-541 (2007) doi:10.1080/05704920701551530
2. N. Stone, C. Kendall, J. Smith, P. Crow and H. Barr: Raman spectroscopy for identification of epithelial cancers. Faraday Discuss, 126, 141-57; discussion 169-83 (2004) doi:10.1039/b304992b
3. I. R. Ramos, A. D. Meade, O. Ibrahim, H. J. Byrne, M. McMenamin, M. McKenna, A. Malkin and F. M. Lyng: Raman spectroscopy for cytopathology of exfoliated cervical cells. Faraday Discuss, 187, 187-98 (2016) doi:10.1039/c5fd00197h
4. J. Ruiz-Chica, M. A. Medina, F. Sanchez-Jimenez and F. J. Ramirez: Fourier transform Raman study of the structural specificities on the interaction between DNA

and biogenic polyamines. Biophys J, 80(1), 443-54 (2001) doi:10.1016/S0006-3495(01)76027-4

5. R. L. Frost, W. Martens, J. T. Kloprogge and Z. Ding: Raman spectroscopy of selected lead minerals of environmental significance. Spectrochimica Acta Part A: Molecular and Biomolecular Spectroscopy, 59(12), 2705-2711 (2003) doi:10.1016/s1386-1425(03)00054-4

6. I. H. Chou, M. Benford, H. T. Beier, G. L. Cote, M. Wang, N. Jing, J. Kameoka and T. A. Good: Nanofluidic biosensing for beta-amyloid detection using surface enhanced Raman spectroscopy. Nano Lett, 8(6), 1729-35 (2008) doi:10.1021/nl0808132

7. S. M. A, R. Crockett, G. Kearn, I. Cherny, E. Gazit, C. S. L and P. J. S: Mechanically functional amyloid fibrils in the adhesive of a marine invertebrate as revealed by Raman spectroscopy and atomic force microscopy. Arch Histol Cytol, 72(4-5), 199-207 (2009) doi:10.1679/aohc.72.199

8. A. L. Fink: Protein aggregation: folding aggregates, inclusion bodies and amyloid. Folding and Design, 3(1), R9-R23 (1998) doi:10.1016/s1359-0278(98)00002-9

9. H. J. Lee, C. Choi and S. J. Lee: Membrane-bound alpha-synuclein has a high aggregation propensity and the ability to seed the aggregation of the cytosolic form. J Biol Chem, 277(1), 671-8 (2002) doi:10.1074/jbc.M107045200

10. C. Mensch, A. Konijnenberg, R. Van Elzen, A.-M. Lambeir, F. Sobott and C. Johannessen: Raman optical activity of humanα-synuclein in intrinsically disordered, micelle-boundα-helical, molten globule and oligomericβ-sheet state. Journal of Raman Spectroscopy, 48(7), 910-918 (2017) doi:10.1002/jrs.5149

11. M. Harris, K. Cilwa, E. A. Elster, B. K. Potter, J. A. Forsberg and N. J. Crane: Pilot study for detection of early changes in tissue associated with heterotopic ossification: moving toward clinical use of Raman spectroscopy. Connect Tissue Res, 56(2), 144-52 (2015) doi:10.3109/03008207.2015.1013190

12. E. W. Blanch, L. A. Morozova-Roche, L. Hecht, W. Noppe and L. D. Barron: Raman optical activity characterization of native and molten globule states of equine lysozyme: comparison with hen lysozyme and bovine alpha-lactalbumin. Biopolymers, 57(4), 235-48 (2000) doi:10.1002/1097-0282(2000)57:4<235::AID-BIP5>3.0.CO;2-H

13. E. W. Blanch, L. Hecht and L. D. Barron: Vibrational Raman optical activity of proteins, nucleic acids, and viruses. Methods, 29(2), 196-209 (2003) doi:10.1016/s1046-2023(02)00310-9

14. J. T. Pelton and L. R. McLean: Spectroscopic methods for analysis of protein secondary structure. Anal Biochem, 277(2), 167-76 (2000) doi:10.1006/abio.1999.4320

15. N. C. Maiti, M. M. Apetri, M. G. Zagorski, P. R. Carey and V. E. Anderson: Raman spectroscopic characterization of secondary structure in natively unfolded proteins: alpha-synuclein. J Am Chem Soc, 126(8), 2399-408 (2004) doi:10.1021/ja0356176

16. M. M. Apetri, N. C. Maiti, M. G. Zagorski, P. R. Carey and V. E. Anderson: Secondary structure of alpha-synuclein oligomers: characterization by raman and atomic force microscopy. J Mol Biol, 355(1), 63-71 (2006) doi:10.1016/j.jmb.2005.10.071

17. F. Zhu, J. Kapitan, G. E. Tranter, P. D. Pudney, N. W. Isaacs, L. Hecht and L. D. Barron: Residual structure in disordered peptides and unfolded proteins from multivariate analysis and ab initio simulation of Raman optical activity data. Proteins, 70(3), 823-33 (2008) doi:10.1002/prot.21593

18. T. Lefevre, M. E. Rousseau and M. Pezolet: Protein secondary structure and orientation in silk as revealed by Raman spectromicroscopy. Biophys J, 92(8), 2885-95 (2007) doi:10.1529/biophysj.106.100339

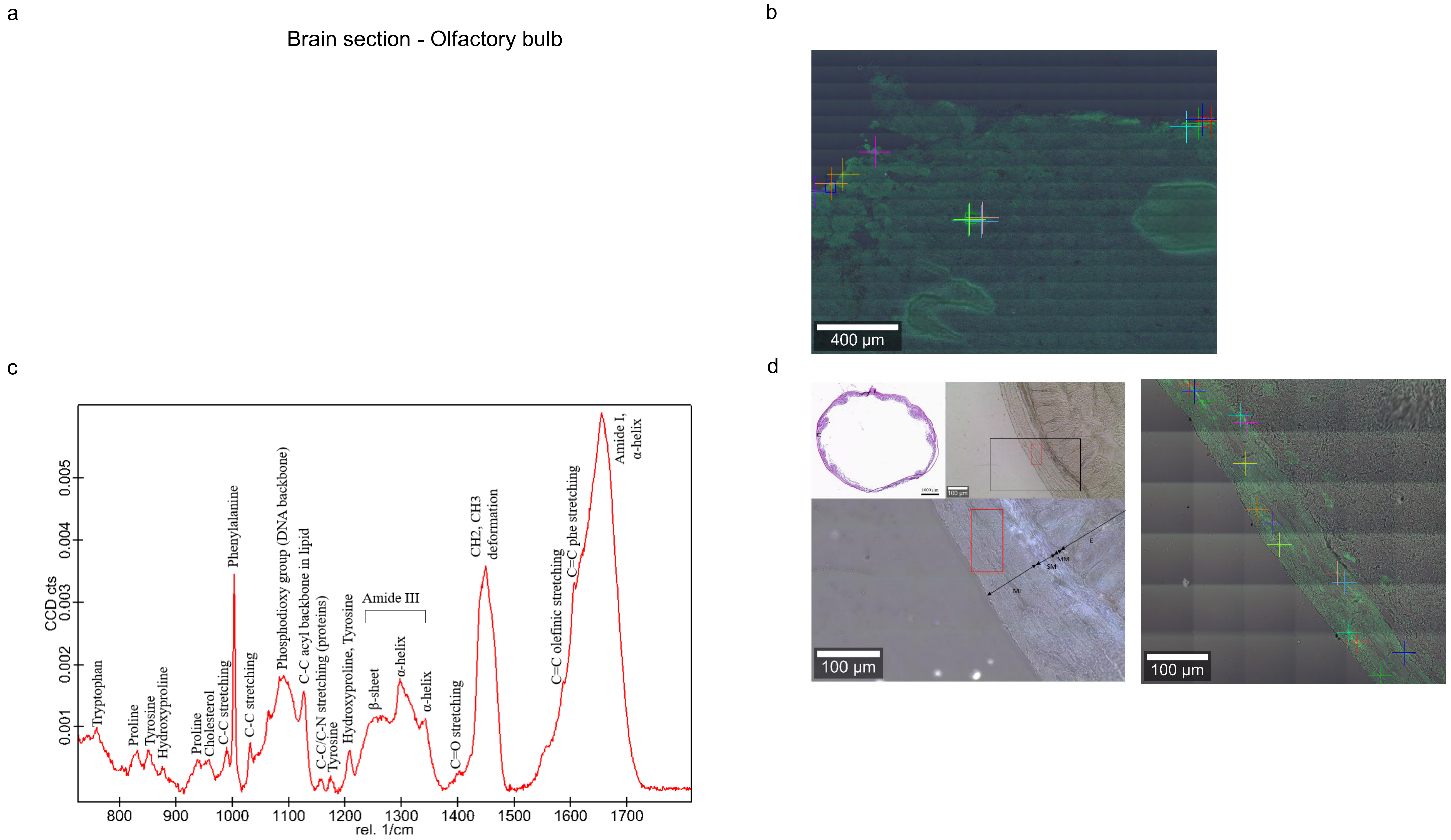

a
Brain section - Olfactory bulb
b
400 µm
c
CCD cts
0.001
0.002
0.003
0.004
0.005
800
900
1000
1100
1200
1300
1400
1500
1600
1700
rel. 1/cm
Tryptophan
Proline
Tyrosine
Hydroxyproline
Proline
Cholesterol
C-C stretching
Phenylalanine
C-C stretching
Phosphodioxy group (DNA backbone)
C-C acyl backbone in lipid
C-C/C-N stretching (proteins)
Tyrosine
Hydroxyproline, Tyrosine
Amide III
β-sheet
α-helix
α-helix
C=O stretching
CH2, CH3
deformation
C=C olefinic stretching
C=C phe stretching
Amide I,
α-helix
d
100 µm
ME
SM
MM
E
100 µm

Suppl. Fig. 1

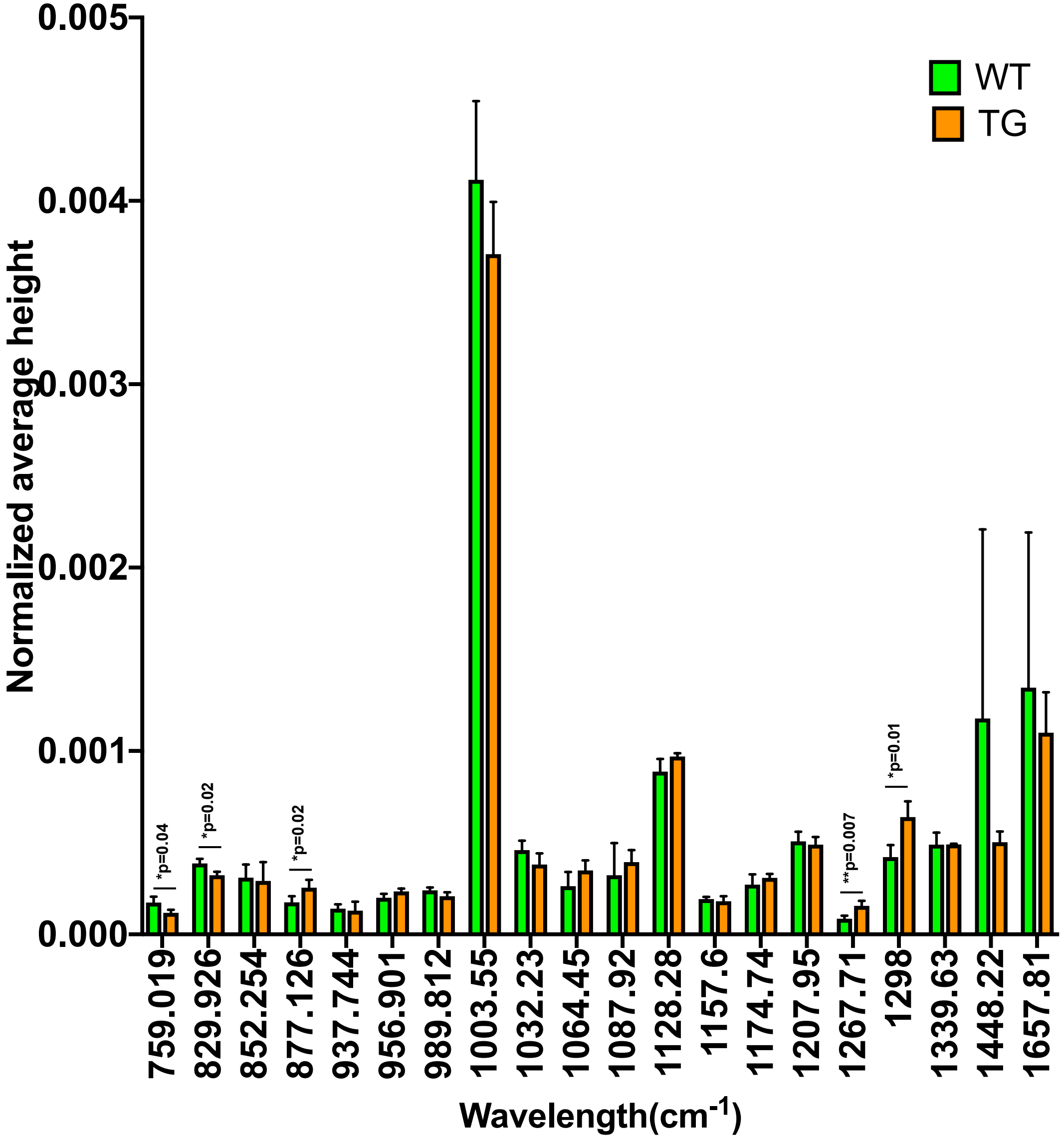
0.005
0.004
0.003
0.002
0.001
0.000
Normalized average height
WT
TG
*p=0.04
*p=0.02
*p=0.02
**p=0.007
*p=0.01
759.019
829.926
852.254
877.126
937.744
956.901
989.812
1003.55
1032.23
1064.45
1087.92
1128.28
1157.6
1174.74
1207.95
1267.71
1298
1339.63
1448.22
1657.81
Wavelength(cm-1)

Suppl. Fig. 2

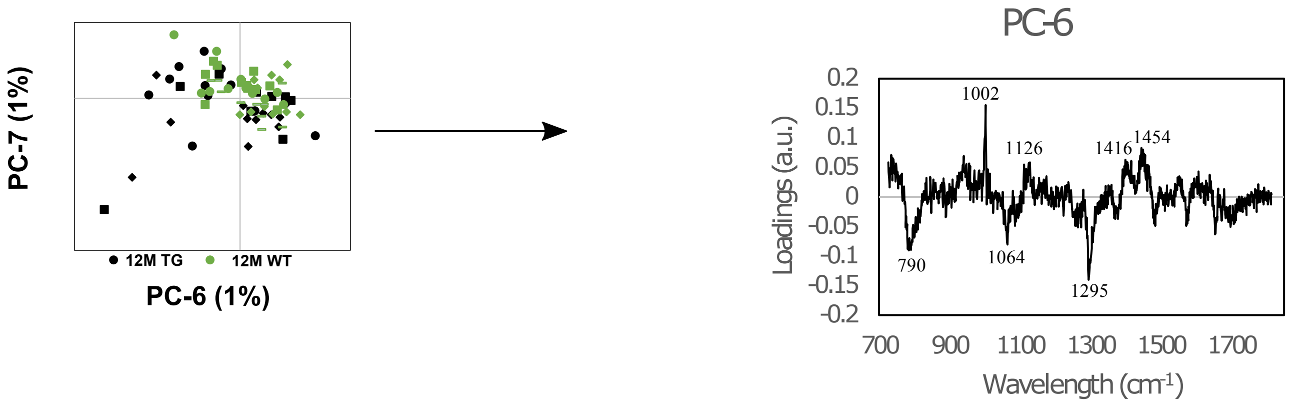

PC-7 (1%)
12M TG
12M WT
PC-6 (1%)
PC-6
0.2
0.15
0.1
0.05
0
-0.05
-0.1
-0.15
-0.2
Loadings (a.u.)
1002
1126
1416
1454
790
1064
1295
700
900
1100
1300
1500
1700
Wavelength (cm-1)

Suppl. Fig. 3

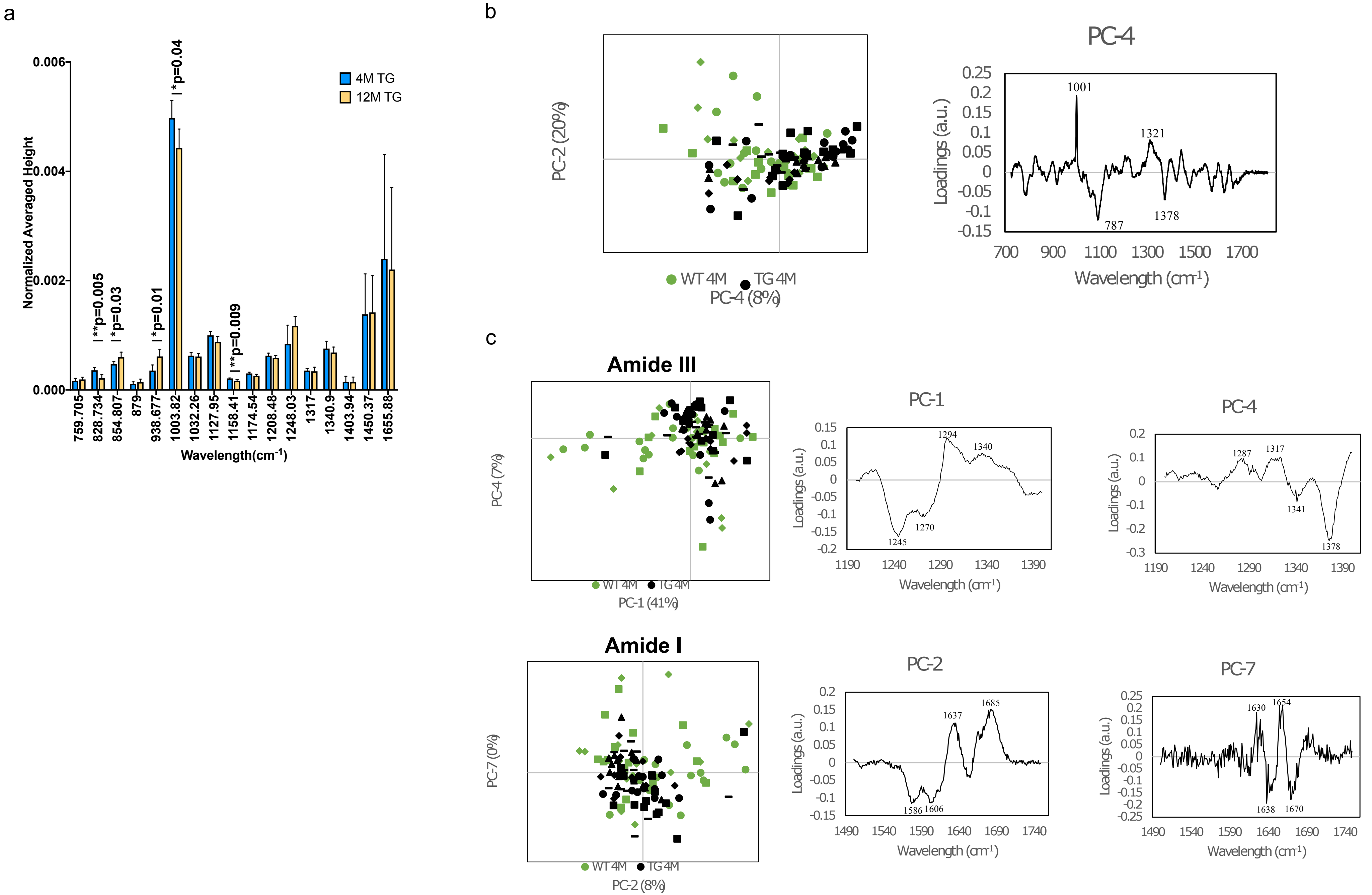
a
Normalized Averaged Height
0.006
0.004
0.002
0.000
4M TG
12M TG
**p=0.005
*p=0.03
*p=0.01
*p=0.04
**p=0.009
759.705
828.734
854.807
879
938.677
1003.82
1032.26
1127.95
1158.41
1174.54
1208.48
1248.03
1317
1340.9
1403.94
1450.37
1655.88
Wavelength(cm-1)
b
PC-2 (20%)
WT 4M
TG 4M
PC-4 (8%)
PC-4
Loadings (a.u.)
Wavelength (cm-1)
1001
1321
787
1378
c
Amide III
PC-4 (7%)
PC-1 (41%)
PC-1
PC-4
1294
1340
1245
1270
1287
1317
1341
1378
Amide I
PC-7 (0%)
PC-2 (8%)
PC-2
PC-7
1637
1685
1586
1606
1630
1654
1638
1670

Suppl. Fig. 4

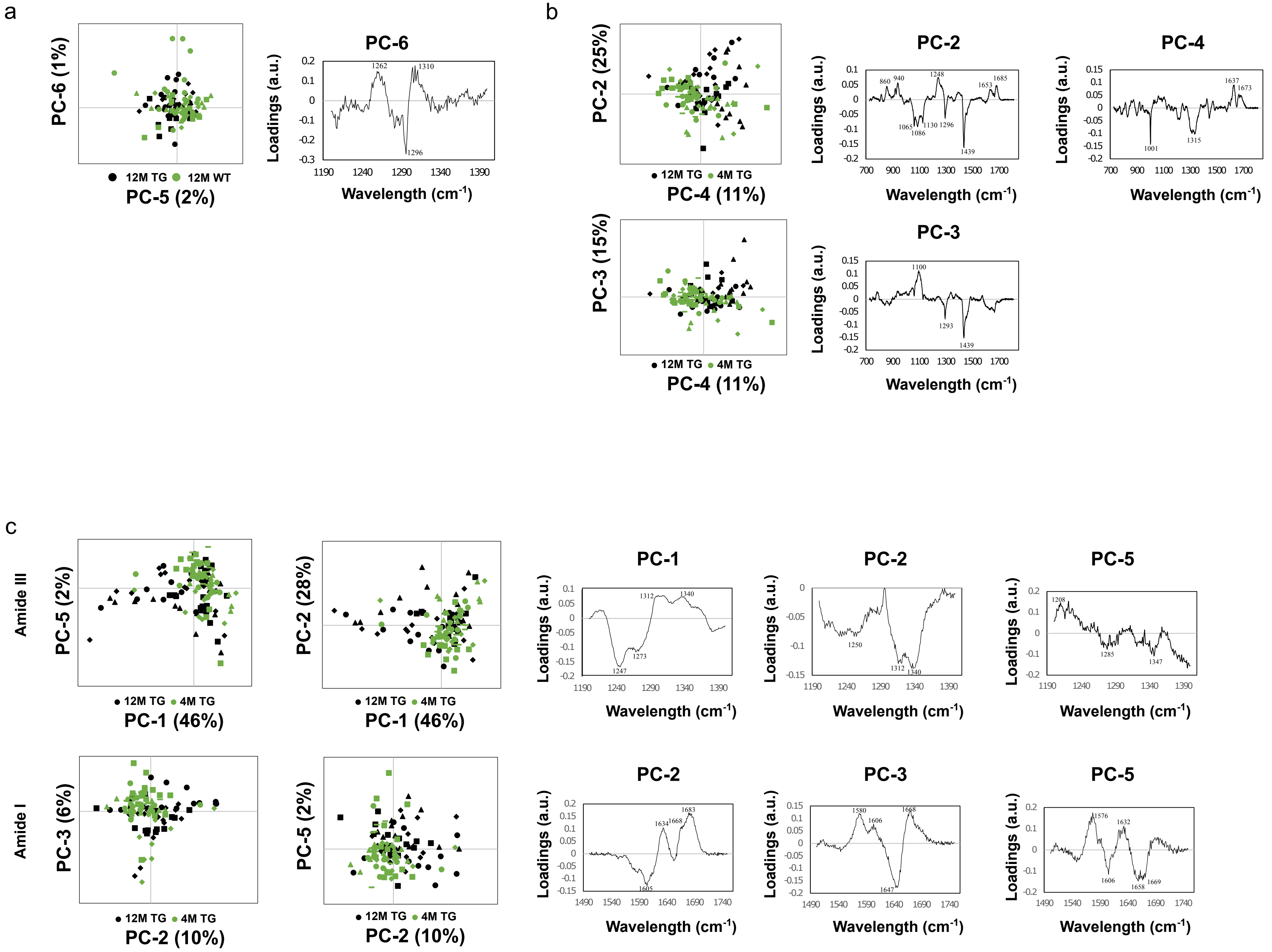

a
PC-6 (1%)
PC-5 (2%)
12M TG
12M WT
PC-6
Loadings (a.u.)
Wavelength (cm-1)
1262
1310
1296
b
PC-2 (25%)
PC-4 (11%)
12M TG
4M TG
PC-2
PC-4
PC-3 (15%)
PC-3
860
940
1248
1653
1685
1065
1086
1130
1296
1439
1637
1673
1001
1315
1100
1293
c
Amide III
PC-5 (2%)
PC-1 (46%)
PC-2 (28%)
PC-1
PC-5
1312
1340
1247
1273
1250
1208
1285
1347
Amide I
PC-3 (6%)
PC-2 (10%)
1634
1668
1683
1605
1580
1606
1647
1576
1632
1658
1669

Suppl. Fig. 5

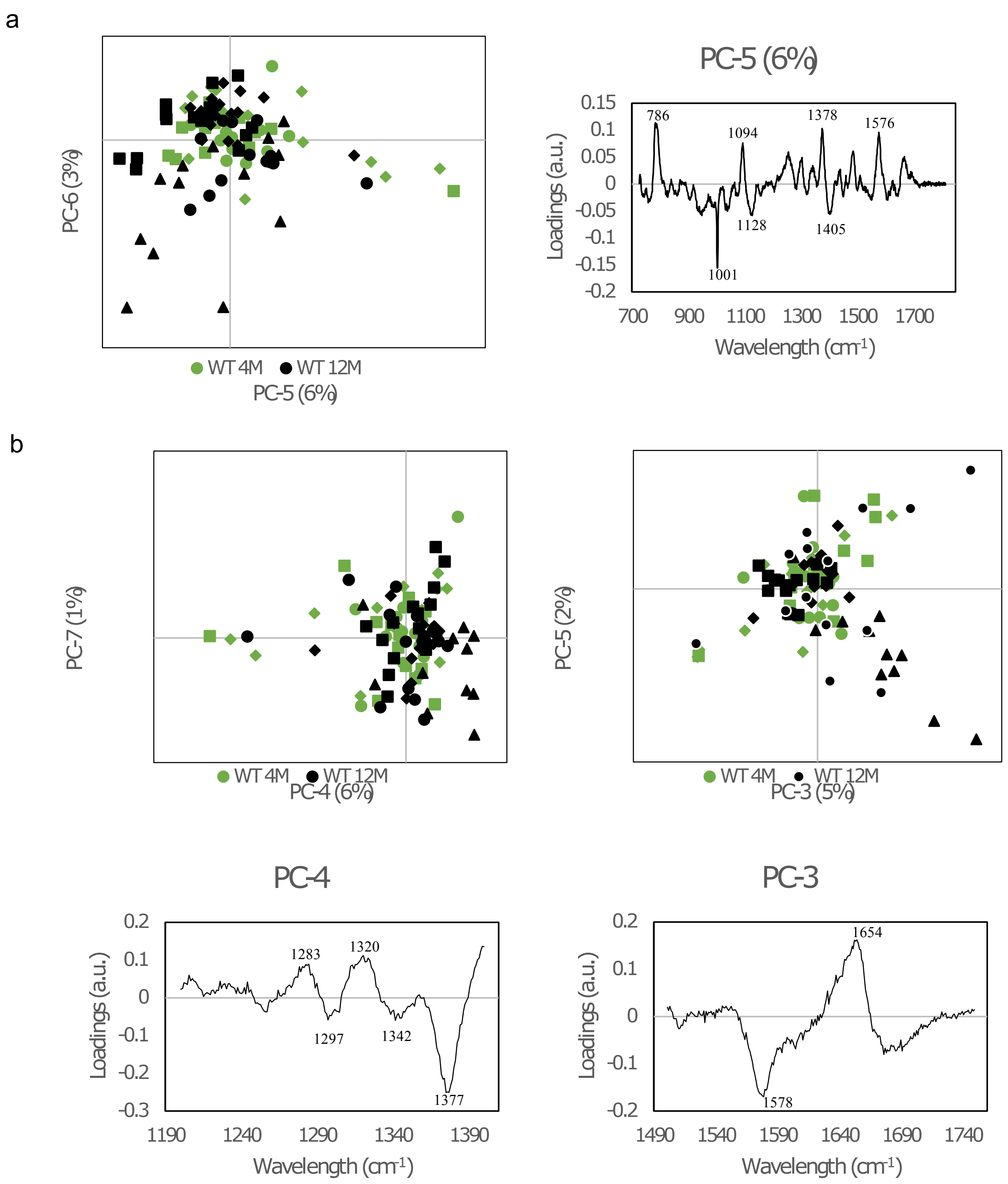

a
PC-6 (3%)
WT 4M
WT 12M
PC-5 (6%)
PC-5 (6%)
Loadings (a.u.)
Wavelength (cm-1)
786
1094
1378
1576
1128
1405
1001
b
PC-7 (1%)
PC-4 (6%)
PC-5 (2%)
PC-3 (5%)
PC-4
1283
1320
1297
1342
1377
PC-3
1654
1578

Suppl. Fig. 6

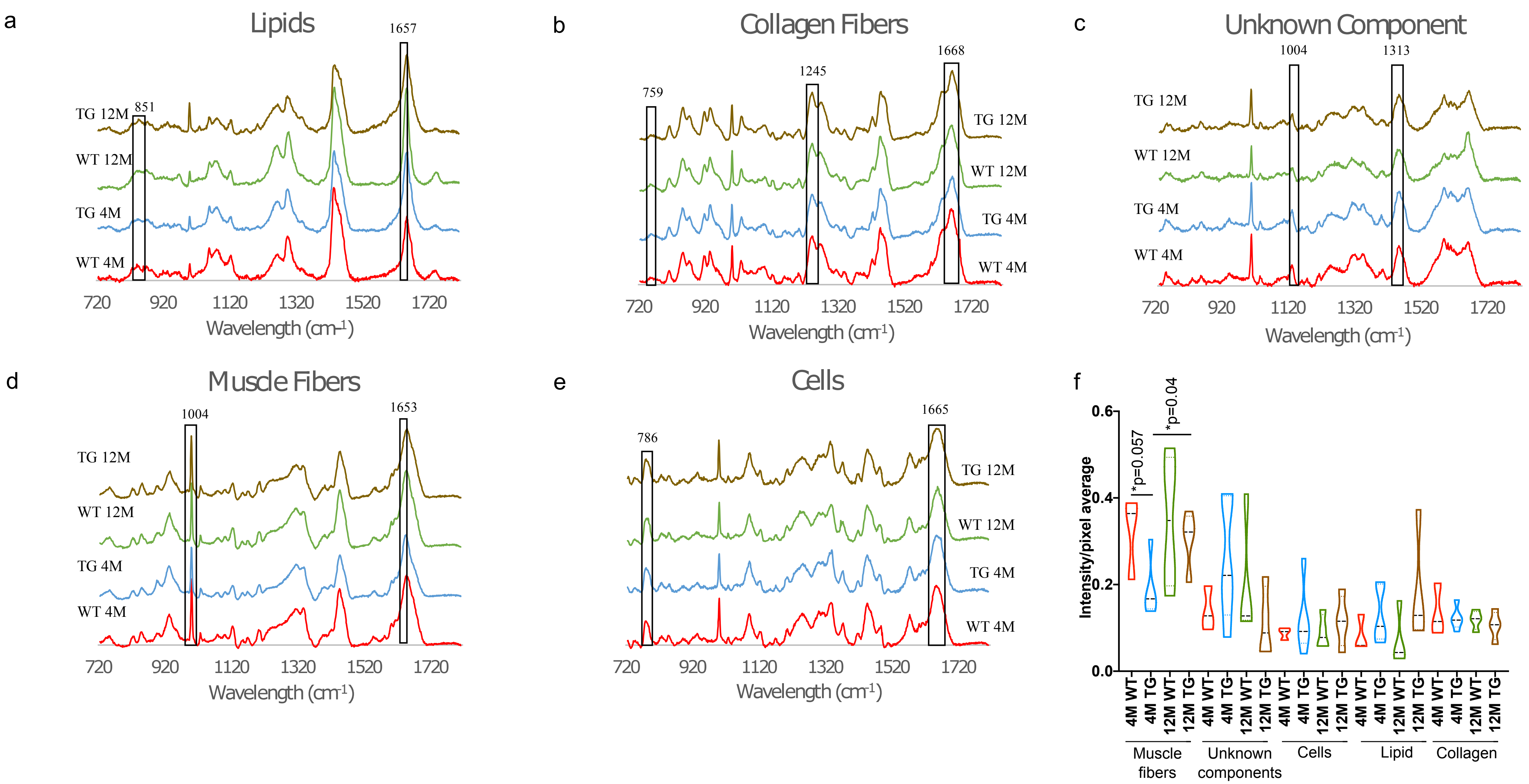
a
Lipids
1657
851
TG 12M
WT 12M
TG 4M
WT 4M
720
920
1120
1320
1520
1720
Wavelength (cm-1)
b
Collagen Fibers
759
1245
1668
TG 12M
WT 12M
TG 4M
WT 4M
Wavelength (cm-1)
c
Unknown Component
1004
1313
TG 12M
WT 12M
TG 4M
WT 4M
Wavelength (cm-1)
d
Muscle Fibers
1004
1653
TG 12M
WT 12M
TG 4M
WT 4M
Wavelength (cm-1)
e
Cells
786
1665
TG 12M
WT 12M
TG 4M
WT 4M
Wavelength (cm-1)
f
Intensity/pixel average
*p=0.057
*p=0.04
0.6
0.4
0.2
0.0
4M WT
4M TG
12M WT
12M TG
Muscle fibers
Unknown components
Cells
Lipid
Collagen

Suppl. Fig. 7
